# Genome-wide response to selection and genetic basis of cold tolerance in rice (*Oryza sativa* L.)

**DOI:** 10.1186/1471-2156-15-55

**Published:** 2014-05-08

**Authors:** Fan Zhang, Xiu-Fang Ma, Yong-Ming Gao, Xian-Bin Hao, Zhi-Kang Li

**Affiliations:** 1 Institute of Crop Sciences/National Key Facility for Crop Gene Resources and Genetic Improvement, Chinese Academy of Agricultural Sciences, Beijing, China; 2 Liaoning Academy of Agricultural Sciences, Shenyang, Liaoning, China

**Keywords:** Selection, Functional genetic units, Non-random association, Epistasis, Hidden genetic diversity

## Abstract

**Background:**

Cold stress is an important factor limiting rice yield in many areas of high latitude and altitude. Considerable efforts have been taken to genetically dissect cold tolerance (CT) in rice using DNA markers. Because of possible epistasis and gene × environment interactions associated with identified quantitative trait loci, the results of these genetic studies have unfortunately not been directly applicable to marker-assisted selection for improved rice CT. In this study, we demonstrated the utility of a selective introgression strategy for simultaneous improvement and genetic dissection of rice seedling CT.

**Results:**

A set of *japonica* introgression lines (ILs) with significantly improved seedling CT were developed from four backcross populations based on two rounds of selection. Genetic characterization of these cold-tolerant ILs revealed two important aspects of genome-wide responses to strong phenotypic selection for rice CT: (1) significant over-introgression of donor alleles at 57 loci in 29 functional genetic units (FGUs) across the rice genome and (2) pronounced non-random associations between or among alleles at many unlinked CT loci. Linkage disequilibrium analyses of the detected CT loci allowed us to construct putative genetic networks (multi-locus structures) underlying the seedling CT of rice. Each network consisted of a single FGU, with high introgression as the putative regulator plus two to three groups of highly associated downstream FGUs. A bioinformatics search of rice genomic regions harboring these putative regulators identified a small set of candidate regulatory genes that are known to be involved in plant stress response.

**Conclusions:**

Our results suggest that CT in rice is controlled by multiple pathways. Genetic complementarity between parental-derived functional alleles at many loci within a given pathway provides an appropriate explanation for the commonly observed hidden diversity and transgressive segregation of CT and other complex traits in rice.

## Background

Rice (*Oryza sativa* L.) is the most important cereal crop and a staple food for more than half the world’s population. Although originating in swampy areas of the tropics, rice is now grown globally in diverse ecologies and thus suffers a wide range of abiotic stresses. Low temperature or cold is a worldwide problem limiting rice yield, particularly in northeast Asia and high mountain areas. Rice crops normally suffer two types of cold stress. During the early rice-crop season, low-temperature stress restrains seedling establishment and plant growth/development; at the reproductive stage, cold induces low fertility and poor grain filling of single-crop rice [1]. In China, cold is responsible for average national yield losses of ~5% and ~2% in the early rice crop of the south and the single-crop rice of most rice growing areas, respectively [2].

Rice genotypes differ considerably in their cold tolerance (CT) [3]. The development of high-yielding, cold-tolerant cultivars is the most efficient way to overcome the problem of low-temperature stress. Because of their generally superior CT compared with that of *indica* rice, subsp. *japonica* accessions are normally used as donors to confer CT when breeding for improved CT [3]. Nevertheless, most *japonica* varieties tend to suffer more frequently from cold because they are distributed in high latitude or high elevation regions. Because of the generally low level of genetic diversity within the *japonica* gene pool, further CT improvement of elite *japonica* cultivars remains a huge breeding challenge [4,5]. Progress with respect to the improvement of rice seedling CT has recently been made using phenotypic selection and a conventional breeding strategy [6]. Considerable efforts have meanwhile been taken to genetically dissect rice CT using DNA markers, resulting in the discovery and mapping of many quantitative trait loci (QTLs) associated with rice CT [7-17]. Two major QTLs for CT, *qCTS4* and *qCTS12*, have been fine-mapped onto rice chromosome 4 and the short arm of chromosome 12, respectively [1,18]. Unfortunately, results from these genetic studies have not been directly applicable to marker-assisted selection for improved rice CT owing to possible epistasis and gene × environment interactions associated with the identified QTLs [19].

We have previously reported several successful applications of a large-scale backcross (BC) breeding strategy to improve abiotic stress tolerance in rice [20-24] as well as a forward genetics strategy for QTL discovery and allelic mining of complex traits using DNA markers and introgression lines (ILs) developed from BC breeding programs [25,26]. In this study, we used a set of *japonica* ILs to demonstrate the utility of this strategy for simultaneous improvement and genetic dissection of rice seedling CT.

## Methods

### Development of cold-tolerant ILs

For the recurrent parent (RP), we used the superior *japonica* restorer line C418, the male parent of many elite *japonica* hybrid cultivars commercially grown in northern China. Four cold-sensitive *indica* lines, Zihui100 (ZH100) from central China, Bg300 from Sri Lanka, Cisanggarung (Cis) from Indonesia, and Manawthukha (MNTH) from Myanmar, were used as donors because the *indica* gene pool is known to contain huge amounts of hidden genetic diversity for abiotic stress tolerance [20,22,23]. C418 was crossed with the donors in the summer of 2001 at the Rice Research Institute of the Liaoning Academy of Agricultural Sciences (LAAS) in Shenyang (41.8°N, 123.4°E) to produce the F_1_ generation. The F_1_ lines were backcrossed with C418 in the Hainan winter nursery during the winter season of 2001 to produce the BC_1_F_1_ population. Twenty-five randomly selected plants from each BC_1_F_1_ line were backcrossed with C418 to produce 25 BC_2_F_1_ lines. From each of the crosses, 25 BC_2_F_1_ lines were planted (15 plants of each line in a single row) at the LAAS Hainan experimental farm in the winter of 2002. Seeds from individual plants of 25 BC_2_F_1_ lines of each cross were bulk-harvested to form a single bulk BC_2_F_2_ population.

The four bulk BC_2_F_2_ populations were screened for seedling CT in the spring of 2003 in LAAS. Approximately 1,000 seeds of each BC_2_F_2_ population were sown in a seedling nursery covered by plastic film on April 3, 15 days earlier than normal planting time, yielding ~800 seedlings from each population. The plastic film was removed at the 2.5-leaf stage, exposing the seedlings for 20 d to outside low temperatures ranging from 6.7 to 23.0°C that severely inhibited the growth of C418. Only 41 seedlings from four BC populations showed normal growth and obviously better CT than C418. These plants were selected and transplanted individually into the field in mid-May. At maturity, seeds from a single panicle were harvested from each of the selected BC progenies as single BC_2_F_3_ lines. Seeds from the 41 BC_2_F_3_ lines were planted as individual lines under normal conditions in the LAAS Hainan winter nursery during the winter of 2003–2004. One or more plants in each of the BC_2_F_3_ lines that had similar or better yield performances than C418 were visually selected and advanced as BC_2_F_4_ ILs. During the following summer and winter of 2004, similar visual selection of BC_2_F_4_ ILs was repeated, resulting in a final set of 177 BC_2_F_6_ ILs derived from the 41 originally selected BC_2_F_2_ plants.

### Progeny testing of selected ILs under natural and controlled low-temperature conditions

Two experiments were performed to confirm the seedling CT of the selected BC_2_F_6_ ILs. In the first experiment, the 177 BC_2_F_6_ ILs and C418 were evaluated under both normal and stress conditions in the LAAS seedling nursery at Shenyang in the early spring of 2005. According to published methods [10,11], soaked seeds of each IL were sown onto a single row plot in the seedling nursery on April 6, 2005. At the 2.5-leaf stage, approximately 15 vigorous seedlings were retained in each plot. The plots were arranged sequentially with three replications for each line. In the low-temperature stress treatment, the plastic film was unveiled to expose the seedlings to the natural low-temperature stress of early spring for 14 d. During this period, the average daily temperature was 12.0°C, and average low and high temperatures were 6.6 ± 2.0°C (ranging from 4.3 to 9.0°C) and 23.7 ± 2.4°C (ranging from 20.9 to 26.8°C), respectively. In the normal control, all plots were completely covered by the plastic film and maintained at an average daily temperature above 20°C. At the end of the treatments, five representative seedlings in each plot under stress or normal conditions were sampled for measurement of their seedling heights (SHs). The sampled seedlings were then dried in an oven at 78°C for 48 h before being measured for seedling dry weight (SDW). Cold response index (CRI) percentages were determined as follows: CRI (%) = (SDW under stress/SDW under normal conditions) × 100.

In the second experiment, the 177 BC_2_F_6_ ILs and their parents were evaluated for seedling CT in a growth chamber at LAAS in 2007. Using a previously reported method [7], well-germinated seeds of each line were sown into a two-row plot in a 65 cm × 44 cm × 14 cm plastic box, six lines per box. The boxes were kept in the growth chamber at a constant day/night temperature of 25°C, a 12-h photoperiod, and a relative humidity at 75–80%. We set up three replications of each IL and six replications of C418. Approximately 30 normal seedlings in each plot were allowed to grow until the three-leaf stage. The growth chamber was then slowly adjusted to a constant day/night temperature of 4°C for 7 d and subsequently slowly adjusted back to a constant day/night temperature of 25°C for 4 d. The number of surviving seedlings in each plot was recorded; seedling survival percentage (SP) was calculated from the average of three replications and used as input data for genetic analysis of seedling CT.

### Genotyping experiments

To reconstruct genotypes of the originally selected BC_2_F_2_ plants, DNA was isolated from bulked fresh leaf tissues of each of the 177 BC_2_F_2:6_ ILs using the CTAB method. We tentatively divided the rice genome into 185 well-distributed bins each representing ~2 Mb physical distance based on the Rice Annotation Project Database IRGSP-1.0 (http://rapdb.dna.affrc.go.jp/). More than 600 rice anchor simple sequence repeat (SSR) markers were used to survey the parental lines, resulting in 298 SSR markers polymorphic between the RP and donors in 137 different bins across the rice genome. The generated markers were used to genotype the 177 selected BC_2_F_6_ ILs (Additional file 1: Table S1). On average, ILs from each BC population were genotyped with 110 polymorphic SSR markers, covering most introgressed donor segments in the ILs.

### Detection of functional genetic units (FGUs) and putative genetic networks (multilocus structures) underlying CT in the selected ILs

Drawing on molecular quantitative genetics theory [26], we define two concepts, functional genetic units (FGUs) and the principle of hierarchy. These concepts are based on the two most common types of functional relationships seen among genes functioning in a signaling pathway affecting complex traits. Hierarchy reflects the one-way functional dependency (FD) of downstream pathway genes on their upstream regulators. A FGU represents the mutual FD among a group of genes functioning at each signaling pathway level; it affects phenotype(s) in the manner of a “house of cards”, i.e., with complete complementarity. Non-random associations (multilocus structures) are predicted to result from these two types of FD between or among unlinked loci within a signaling pathway. Furthermore, upstream FGUs (regulators) in a signaling pathway affecting complex traits are expected to have larger phenotypic effects and thus respond more strongly to selection than downstream FGUs. In our selection experiment, alleles at segregating regulatory loci affecting the target trait were thus expected, in response to selection, to show greater shifts in introgression frequencies (IFs) of the functional genotypes (defined as either one of the two homozygotes or the heterozygote containing the functional allele at the identified loci).

Using the progeny testing data observed from the 177 ILs under controlled low-temperature conditions, contrast tests were performed using SAS PROC GLM [27] to compare differences in SP between ILs and C418. As a result, 30 BC_2_F_2:6_ ILs were identified that had significantly higher SPs than C418. Based on the above-described concepts, a FGU could comprise either single loci showing significant excess introgression or an association group (AG) of *r* (where *r* ≥ 2) unlinked but perfectly associated loci exhibiting equal introgression in the cold-tolerant ILs selected from each BC population. To detect FGUs and multilocus structures (putative genetic networks) associated with CT in rice, we conducted statistical tests using the genotypic data from the 30 confirmed cold-tolerant BC_2_ progeny. First, χ^*2*^ tests were performed to detect whether allelic and genotypic frequencies at individual loci across the genome in the CT ILs from each BC population deviated significantly from Mendelian expectations. Those loci showing significant over-introgression were considered to be putative CT loci [25]. To minimize the probability of false positives, we used a conservative threshold of *P* < 0.001 for claiming a putative CT locus. Second, linkage disequilibrium (LD) analyses [28] were performed to detect non-random associations or AGs between or among unlinked loci showing excess introgression. For each AG consisting of *r* (where *r* ≥ 2) unlinked loci exhibiting equal levels of excess donor alleles co-introgression in confirmed cold-tolerant ILs from each population, *r ·* (*r–*1)/2 significant pairwise associations would be expected to exist between the *r* loci. An AG was considered to be significant on the basis of the following thresholds *P* ≤ 0.005 and D*'* = 1.0 for each of the *r ·* (*r–*1)/2 pairwise associations. To reveal the multilocus structure or putative genetic network underlying CT, pairwise gametic LD analyses were performed to characterize the relationships between alleles at all FGUs detected in the cold-tolerant ILs from each BC population. Using the genotypic data of the ILs from each BC population, we calculated the LD statistic [28]${\hat{D}}_{\mathrm{AB}}={\tilde{p}}_{\mathrm{AB}}-{\tilde{p}}_{A}{\tilde{p}}_{B}$, where ${\tilde{p}}_{\mathrm{AB}}$, ${\tilde{p}}_{A}$, and ${\tilde{p}}_{B}$ were the frequencies of co-introgressed functional genotype AB and functional genotypes at FGU A and FGU B, respectively. Here, a functional genotype was either the donor homozygote or the heterozygote. For significance testing of ${\hat{D}}_{\mathrm{AB}}$, we used the test statistic $\chi_{\mathrm{AB}}^{2}=\frac{2n{\hat{D}}_{\mathrm{AB}}^{2}}{{\tilde{p}}_{A}\left(1-{\tilde{p}}_{A}\right){\tilde{p}}_{B}\left(1-{\tilde{p}}_{B}\right)}$ , where *n* was the sample size and the normalized ${\hat{D}}_{\mathrm{AB}}$ was estimated as ${\hat{D}}_{\mathrm{AB}}^{'}=\frac{{\hat{D}}_{\mathrm{AB}}}{{\hat{D}}_{\text{max}}}$, with ${\hat{D}}_{\max}=\min\left({\tilde{p}}_{A}{\tilde{p}}_{\bar{B}},{\tilde{p}}_{\bar{A}}{\tilde{p}}_{B}\right)$ if ${\hat{D}}_{\mathrm{AB}}>0$, else ${\hat{D}}_{\text{max}}=\min\left({\tilde{p}}_{A}{\tilde{p}}_{B},{\tilde{p}}_{\bar{A}}{\tilde{p}}_{\bar{B}}\right)$. A multilocus genetic network containing all detected FGUs in the confirmed cold-tolerant ILs was then constructed in two steps. First, all FGUs detected in ILs from a single population were divided into major groups based on the LD results. The criterion used for delineating these groups was that individual FGUs of different IFs within each group were all significantly and positively associated, with $\hat{D}{s_{\mathrm{AB}}}^{'}$= 1.0, and were either independent or negatively associated with FGUs in different groups. Second, all FGUs within a given group were connected, forming multiple layers based on the principle of hierarchy according to their progressively reduced functional genotype frequencies and inclusive relationships [26]. In addition, to confirm the detected loci, we used the two-sample test for proportions in SAS to compare the IF at each identified CT locus between two segregating groups of BC_2_F_6_ ILs with contrasting CT phenotypes, either high SP or low SP, all derived from the same selected BC_2_F_2_ plants [27]. Detection of a significant difference between these two segregating BC_2_F_6_ groups would verify the CT FGUs determined using the previous method. Finally, a bioinformatics search based on the RGAP 7 database and information in Rice Genome Annotation Project data libraries (http://rice.plantbiology.msu.edu) was performed to identify possible positional candidate genes for CT in those genomic regions (mapped markers) where important CT FGUs were detected.

## Results

### Development of ILs with significantly improved CT

From the ~3,200 BC_2_F_2_ plants in the four populations subjected to 20 d of natural low-temperature stress, 41 BC_2_F_2_ plants were selected. The number of selected plants ranged from 7 in the C418/MNTH population to 15 in the C418/CG population, with an average selection intensity of 1.3% (Table 1).

**Table 1:** **Results of screening for seedling cold tolerance (CT) of four BC**_**2**_**F**_**2 **_**populations derived from crosses between C418 (*japonica*) and four *indica *** **donors and introgression patterns of the resultant 30 verified cold-tolerant introgression lines**

**Population code**	**Donor**	**N**_**1**_^**1**^	**N**_**2**_^**1**^	**SI (%)**^**1**^	**N**_**3**_^**1**^	**B**^**2**^	**H**^**2**^	**IF**^**2**^
A	Zihui100	800	8	1.0	6	0.086	0.179	0.175
B	Bg300	800	11	1.4	6	0.035	0.373	0.222
C	Cisanggarung	800	15	1.9	13	0.086	0.289	0.231
D	Manawthukha	800	7	0.9	5	0.018	0.315	0.176
	Average	800	10.3	1.3	7.5	0.056	0.289	0.201

^1^N_1_ is the original size of the BC_2_F_2_ population used for screening seedling CT. N_2_ is the number of surviving plants selected from each population after exposure at the seedling stage to a 20-d cold treatment. N_3_ is the number of selected BC progeny with significantly improved CT as confirmed by progeny testing of their derived BC_2_F_6_ ILs under 7-d cold treatment in a phytotron at the seedling stage. SI is the selection intensity.

^2^B, H and IF are frequencies of the donor homozygote, heterozygote, and donor introgression, respectively, in the selected CT ILs.

Progeny testing of the 177 derived BC_2_F_2:6_ ILs to confirm their CT under more severe cold stress in a growth chamber revealed that 68 BC_2_F_6_ ILs derived from 30 of the 41 selected BC_2_F_2_ plants had significantly higher SPs and CRI values than C418 (Table 2; Additional file 2: Figure S1). Average seedling SP of the BC_2_F_2:6_ ILs was ~3.3 times higher than that of C418, ranging from 1.6 times higher for the 62 ILs in the C418/Bg300 population to 4.7 times higher for the 53 ILs in the C418/CG population. Compared with C418, the ILs had significantly increased SH and SDW CRIs under natural low-temperature stress, respectively 6% and 18% higher (Additional file 2: Figure S1). Thus, 30 of the 41 selected ILs were confirmed to have significantly improved CT over that of C418, including 6 lines each from the C418/ZH100 and C418/Bg300 populations, 13 lines from the C418/CG population, and 5 lines from the C418/MNTH population (Table 2).

**Table 2:** **Results of progeny testing of 41 cold-tolerant BC**_**2**_**F**_**2 **_**individuals from four BC populations based on survival percentages (SPs) of their derived BC**_**2**_**F**_**2:6 **_**progeny evaluated under conditions of 4°C for 7 d in a growth chamber**

**Pop.**^**1**^	**BC**_**2**_**F**_**2 **_**selected ID**	**BC**_**2**_**F**_**2 **_**verified ID**	**# of BC**_**2**_**F**_**2:6 **_**ILs**	***Z*-test**^**2**^	**SP ± S.D. (%)**^**3**^	**Cold response index**^**4**^	
						**SH ± SD (%)**	**SDW ± SD (%)**
ZH100	031 K85	A-IL1	1		30.0 ± n.a.**	81.0 ± n.a.	75.4 ± n.a.
	031 K86	A-IL2	2		46.1 ± 25.6**	80.3 ± 11.0	75.0 ± 16.0
	031 K89	A-IL3	5	√	23.7 ± 18.4*	88.5 ± 23.4	88.5 ± 35.3
	031 K90	A-IL4	2		40.3 ± 0.2**	100.7 ± 20.7	102.4 ± 10.6
	031 K92	A-IL5	5	√	41.4 ± 26.7**	91.7 ± 10.2	101.9 ± 33.8
	031 K97	A-IL6	1		66.0 ± n.a.**	75.9 ± n.a.	59.4 ± n.a.
	031 K96	-	1		9.7 ± n.a.	95.5 ± n.a.	82.4 ± n.a.
	031 K101	-	1		10.0 ± n.a.	70.9 ± n.a.	82.5 ± n.a.
Bg300	031 K188	B-IL1	26	√	66.6 ± 30.2**	87.5 ± 12.4	114.1 ± 30.8
	031 K187	B-IL2	10		23.8 ± 9.5*	83.8 ± 9.4	113.6 ± 31.6
	031 K190	B-IL3	5		40.2 ± 4.0**	82.5 ± 9.7	134.2 ± 30.6
	031 K195	B-IL4	11	√	20.7 ± 22.3*	76.3 ± 7.0	97.4 ± 19.0
	031 K196	B-IL5	8	√	17.2 ± 22.2*	72.1 ± 6.1	97.7 ± 20.7
	031 K189	B-IL6	2		41.5 ± 0.8**	71.7 ± 7.9	90.9 ± 3.8
	031 K186	-	1		0.0 ± n.a.	78.0 ± n.a.	82.1 ± n.a.
	031 K191	-	6		1.2 ± 1.8	86.1 ± 7.0	117.5 ± 35.4
	031 K192	-	1		0.0 ± n.a.	82.2 ± n.a.	90.8 ± n.a.
	031 K193	-	5		17.2 ± 13.4	88.6 ± 15.1	117.5 ± 17.7
	031 K194	-	6		0.0 ± 0.0	81.6 ± 12.2	98.5 ± 14.0
Cis	031 K155	C-IL1	7	√	30.2 ± 22.2**	81.6 ± 16.9	88.3 ± 30.7
	031 K156	C-IL2	14	√	22.2 ± 23.6*	73.5 ± 13.1	77.1 ± 17.5
	031 K158	C-IL3	11	√	46.5 ± 35.5**	79.4 ± 8.6	74.1 ± 19.7
	031 K159	C-IL4	1		95.4 ± n.a.**	90.2 ± n.a.	106.0 ± n.a.
	031 K160	C-IL5	5		82.2 ± 20.7**	78.8 ± 7.7	86.5 ± 26.7
	031 K161	C-IL6	2		85.1 ± 0.3**	89.9 ± 5.8	113.7 ± 27.3
	031 K162	C-IL7	3		66.0 ± 4.3**	84.0 ± 4.3	79.0 ± 30.9
	031 K163	C-IL8	1		74.6 ± n.a.**	76.3 ± n.a.	59.2 ± n.a.
	031 K165	C-IL9	2		79.2 ± 23.9**	79.1 ± 20.8	80.4 ± 16.8
	031 K168	C-IL10	1		68.6 ± n.a.**	67.3 ± n.a.	73.8 ± n.a.
	031 K170	C-IL11	1		78.2 ± n.a.**	96.0 ± n.a.	90.1 ± n.a.
	031 K171	C-IL12	4		69.5 ± 16.3**	76.5 ± 5.2	92.3 ± 17.0
	031 K172	C-IL13	1		53.3 ± n.a.**	72.4 ± n.a.	92.4 ± n.a.
	031 K166	-	1		14.3 ± n.a.	91.1 ± n.a.	55.9 ± n.a.
	031 K157	-	1		15.8 ± n.a.	65.1 ± n.a.	82.0 ± n.a.
MNTH	031 K5	D-IL1	3		67.6 ± 28.2**	85.7 ± 22.6	166.9 ± 55.7
	031 K12	D-IL2	2		52.8 ± 3.2**	65.5 ± 2.7	89.7 ± 6.9
	031 K20	D-IL3	6		49.8 ± 12.9**	70.9 ± 7.5	84.8 ± 3.4
	031 K21	D-IL4	2		46.6 ± 27.5**	63.9 ± 15.1	88.3 ± 37.9
	031 K22	D-IL5	6	√	43.4 ± 16.0**	78.3 ± 6.4	90.6 ± 19.2
	031 K8	-	1		7.8 ± n.a.	74.8 ± n.a.	90.4 ± n.a.
	031 K15	-	3		11.9 ± 5.0	63.6 ± 21.2	41.0 ± 0.6
C418					13.1 ± 7.1	73.9 ± n.a.	77.6 ± n.a.

^1^ZH100, Cis, and MNTH represent Zihui100, Cisanggarung, and Manawthukha, respectively.

^2^“√” indicates that the two groups of BC_2_F_2:6_ ILs derived from the same BC_2_F_2_ individual had distinct CT phenotypes according to the two-sample proportion test.

^3^* and ** indicate that the SPs of BC_2_F_2:6_ ILs were significantly higher than the average SP of recurrent parent C418 at *P* < 0.05 and 0.01, respectively, according to the contrast test.

^4^SH and SDW are seedling height (in cm) and seedling dry weight (in g), respectively, under natural low-temperature stress.

### Genome-wide responses to selection for CT and detection of FGUs for CT

Strong phenotypic selection for CT resulted in significant over-introgression of the donor segments in the 30 verified cold-tolerant BC_2_F_2_ ILs. On average, donor introgression was 0.201 in the cold-tolerant BC_2_F_2_ ILs (Table 1), 160% more than the value of 0.125 expected in a BC_2_ population. This additional introgression was primarily the result of genome-wide excess heterozygosity. The BC_2_ ILs had an average donor homozygote frequency of 0.056, slightly lower than expected, and an average heterozygote frequency of 0.289, which was 2.3 times higher than the expected value (Table 1).

**Figure 1:**
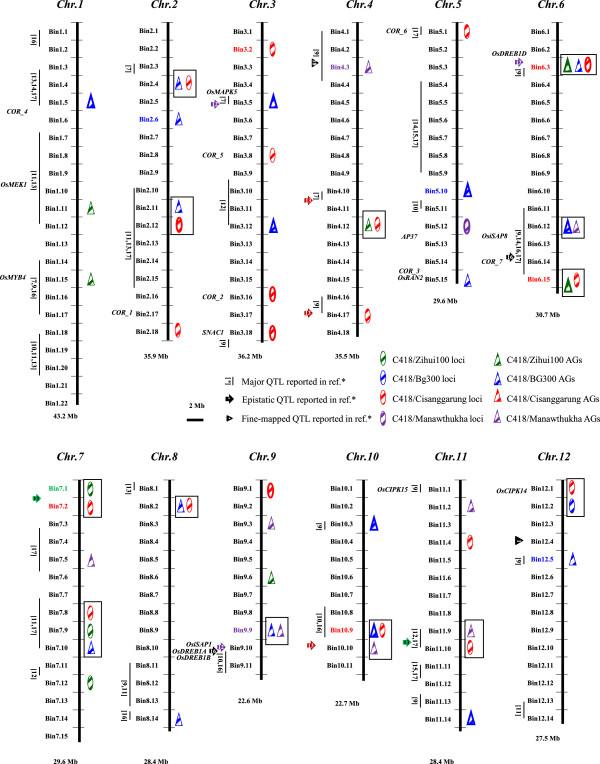
**Genomic distribution into 48 bins of 57 loci in 29 functional genetic units (FGUs) underlying seedling cold tolerance (CT) in 30 introgression lines (ILs) from four BC**_**2**_**F**_**2 **_**populations of rice.** Boxes on the right side of each chromosome are FGUs detected in cold-tolerant ILs; symbols on the left are main-effect QTLs and epistatic QTLs associated with CT previously reported in other rice populations (Table 3; Additional file 1: Table S4). CT-related genes harbored in or near FGU regions are shown on the left side of each chromosome. Boxes with thicker outlines indicate FGUs verified by comparison of introgression frequencies between two groups of BC_2_F_2:6_ ILs with contrasting CT phenotypes derived from the same BC_2_F_2_ plants. Colored bins indicate FGUs of high introgression that are very likely to be upstream regulatory genes in the genetic networks (Figure 2A–D) according to molecular quantitative genetics theory [26]. Regions in rectangular boxes represent linked bins detected in multiple populations.

χ^2^ tests at individual loci and LD analyses using the SSR genotypic data identified 57 loci in 29 FGUs (17 single loci and 12 AGs) across the rice genome at which highly significant over-introgression of the donor alleles was detected in the selected cold-tolerant ILs. The number of identified loci ranged from 9 loci in 5 FGUs in the C418/ZH100 population to 20 loci in 15 FGUs in the C418/Cis population (Table 3; Additional file 1: Table S2). The average IF of the donor alleles at the 57 CT loci was 0.469, or 3.75 times as much as the expected value of 0.125 (Table 3). These FGUs were distributed in 48 bins (~25%) across the rice genome (Figure 1; Table 3). Of the 41 genomic regions in which CT loci were detected, 15 (37%) were verified by comparing donor frequencies of BC_2_F_2:6_ ILs segregating into two contrasting CT groups derived from the same BC_2_F_2_ plants (Figure 1; Table 3). These confirmed loci were located in bins 1.5, 2.11–2.12, 3.5, 3.12, 3.16, 3.18, 5.10, 5.12, 6.3, 6.12, 6.15, 9.1, 10.3, 10.9–10.10, and 11.14 (Table 3).

**Table 3:** **Genomic information for 29 functional genetic units (FGUs) (17 single loci and 12 association groups or AGs) for seedling cold tolerance (CT) detected by χ**^***2 ***^**tests (single loci) and multi-locus linkage disequilibrium analyses in 30 cold-tolerant introgression lines (ILs) selected from four BC**_**2**_**F**_**2 **_**populations derived from crosses between the recurrent parent (C418) and four donors (ZH100, Bg300, Cis, and MNTH)**

**Population (code)**	**χ**^***2 ***^**test**						***Z*-test**^**4**^		**QTL ref.**
	**AG**^**1**^	**Bin**	**Marker**	**IF**^**2**^	**FG**^**3**^	***P*-value**	**BC**_**2**_**F**_**2 **_**individual**	***P*-value**	
C418/ZH100 (A)		7.1	RM4584	0.917	1.000	3.4 × 10^−14^			
	*AG*_*A1*_	1.11	RM1196	0.583	0.833	2.2 × 10^−4^			[11,13]
	*AG*_*A1*_	1.15	RM1297	0.500	0.833	1.1 × 10^−4^			[7,9,16]
	*AG*_*A1*_	4.12	RM2521	0.750	0.833	5.1 × 10^−9^			[7]
	*AG*_*A1*_	9.6	RM1896	0.750	0.833	5.1 × 10^−9^			
	*AG*_*A2*_	6.3	RM5754	0.417	0.667	8.7 × 10^−3^	A-IL5	0.0228	[9]
	*AG*_*A2*_	6.15	RM5463	0.500	0.667	4.5 × 10^−3^	A-IL5	0.0228	[9,14,16,17]
		7.9	RM6835	0.500	0.833	4.5 × 10^−3^			[11,17]
		7.12	RM5847	0.583	0.833	2.2 × 10^−4^			
C418/Bg300 (B)	*AG*_*B1*_	2.6	RM324	0.500	1.000	7.6 × 10^−10^			
	*AG*_*B1*_	5.10	RM164	0.583	1.000	3.1 × 10^−7^	B-IL5	0.0416	[10]
	*AG*_*B1*_	12.5	RM101	0.583	1.000	3.1 × 10^−7^			[1,9]
	*AG*_*B2*_	2.4	RM145	0.500	0.833	1.1 × 10^−4^			[7]
	*AG*_*B2*_	2.11	RM262	0.500	0.833	1.1 × 10^−4^			[11,13,17]
	*AG*_*B2*_	7.10	RM432	0.417	0.833	1.0 × 10^−6^			[11,17]
	*AG*_*B2*_	8.2	RM38	0.417	0.833	1.0 × 10^−6^			[13]
	*AG*_*B3*_	3.5	RM7	0.500	0.833	1.1 × 10^−4^	B-IL1	0.0368	[7]
	*AG*_*B3*_	6.3	RM253	0.583	0.833	2.2 × 10^−4^			[9]
	*AG*_*B3*_	9.9	RM257	0.500	0.833	1.1 × 10^−4^			[10,16]
	*AG*_*B4*_	1.5	RM579	0.333	0.667	3.1 × 10^−4^	B-IL4	0.0289	[13,14,17]
	*AG*_*B4*_	3.12	RM6832	0.333	0.667	3.1 × 10^−4^	B-IL5	0.0416	[12]
	*AG*_*B4*_	5.15	RM334	0.333	0.667	3.1 × 10^−4^			
	*AG*_*B4*_	6.12	RM275	0.333	0.667	3.1 × 10^−4^	B-IL5	0.0072	[9,14,16,17]
	*AG*_*B4*_	10.3	RM216	0.333	0.667	3.1 × 10^−4^	B-IL4/B-IL5	0.0289/0.0072	[9]
	*AG*_*B4*_	10.9	RM1375	0.333	0.667	3.1 × 10^−4^	B-IL4	0.0480	[10,16]
	*AG*_*B4*_	11.14	RM224	0.333	0.667	3.1 × 10^−4^	B-IL4/B-IL5	0.0082/0.0416	
		8.14	RM447	0.333	0.667	3.1 × 10^−4^			[9,11,16]
		12.2	RM247	0.333	0.667	3.1 × 10^−4^			
C418/Cis (C)		7.2	RM5711	0.538	1.000	2.1 × 10^−17^			
	*AG*_*C1*_	6.15	RM3307	0.577	0.846	8.3 × 10^−9^			[9,14,16,17]
	*AG*_*C1*_	10.9	RM1873	0.577	0.846	8.3 × 10^−9^			[10,16]
		12.1	RM6973	0.385	0.692	4.7 × 10^−7^			
		2.18	RM3850	0.308	0.615	5.7 × 10^−7^			
		6.3	RM5754	0.539	0.923	2.3 × 10^−12^	C-IL2	0.0041	[9]
	*AG*_*C2*_	3.8	RM6959	0.423	0.769	3.5 × 10^−9^			
	*AG*_*C2*_	3.16	RM3199	0.423	0.769	3.5 × 10^−9^	C-IL1	0.0228	
	*AG*_*C2*_	9.1	RM3609	0.538	0.769	3.6 × 10^−7^	C-IL2	0.0055	
		4.17	RM6238	0.462	0.692	1.0 × 10^−7^			[9]
		4.12	RM2521	0.308	0.538	1.0 × 10^−3^			[7]
		11.10	RM5349	0.308	0.615	5.7 × 10^−7^			[12,17]
		3.2	RM3126	0.654	0.923	3.7 × 10^−11^			
		3.18	RM3329	0.346	0.538	3.1 × 10^−5^	C-IL3	0.0283	[9]
	*AG*_*C3*_	7.8	RM5875	0.500	0.769	4.8 × 10^−7^			[11,17]
	*AG*_*C3*_	8.2	RM1148	0.462	0.769	1.0 × 10^−7^			[13]
	*AG*_*C4*_	2.12	RM3688	0.346	0.615	3.1 × 10^−5^	C-IL2	0.0055	[11,13,17]
	*AG*_*C4*_	11.4	RM3133	0.308	0.615	5.7 × 10^−7^			
		5.1	RM1200	0.308	0.615	5.7 × 10^−7^			[17]
		2.4	RM5459	0.462	0.615	7.8 × 10^−5^			[7]
C418/MNTH (D)	*AG*_*D1*_	4.3	RM3658	0.700	1.000	1.5 × 10^−5^			[9,18]
	*AG*_*D1*_	9.9	RM6235	0.500	1.000	2.5 × 10^−8^			[10,16]
	*AG*_*D2*_	6.12	RM6298	0.400	0.800	3.0 × 10^−5^			[9,14,16,17]
	*AG*_*D2*_	7.5	RM5436	0.400	0.800	3.0 × 10^−5^			[17]
	*AG*_*D2*_	9.3	RM5899	0.600	0.800	7.3 × 10^−4^			
	*AG*_*D2*_	10.10	RM1146	0.600	0.800	7.3 × 10^−4^			[10,16]
	*AG*_*D2*_	11.2	RM1812	0.400	0.800	3.0 × 10^−5^			
	*AG*_*D2*_	11.9	RM1355	0.400	0.800	3.0 × 10^−5^			[12,17]
		5.12	RM5970	0.400	0.800	3.0 × 10^−5^	D-IL5	0.0416	[10]

^1^An AG is defined as a group of unlinked but perfectly associated loci showing equal introgression in selected cold-tolerant ILs from each BC population as detected by linkage disequilibrium analysis [28].

^2^IF = introgression (donor) allelic frequency.

^3^FG is the frequency of functional genotypes (donor homozygote and the heterozygote).

^4^Two-sample *Z* tests to compare the proportions of two groups of BC_2_F_6_ ILs derived from the same BC_2_F_2_ individual but with significantly different CT phenotypes (SP) was performed using SAS [27].

### Putative genetic networks (multi-locus structures) underlying CT

**Figure 2:**
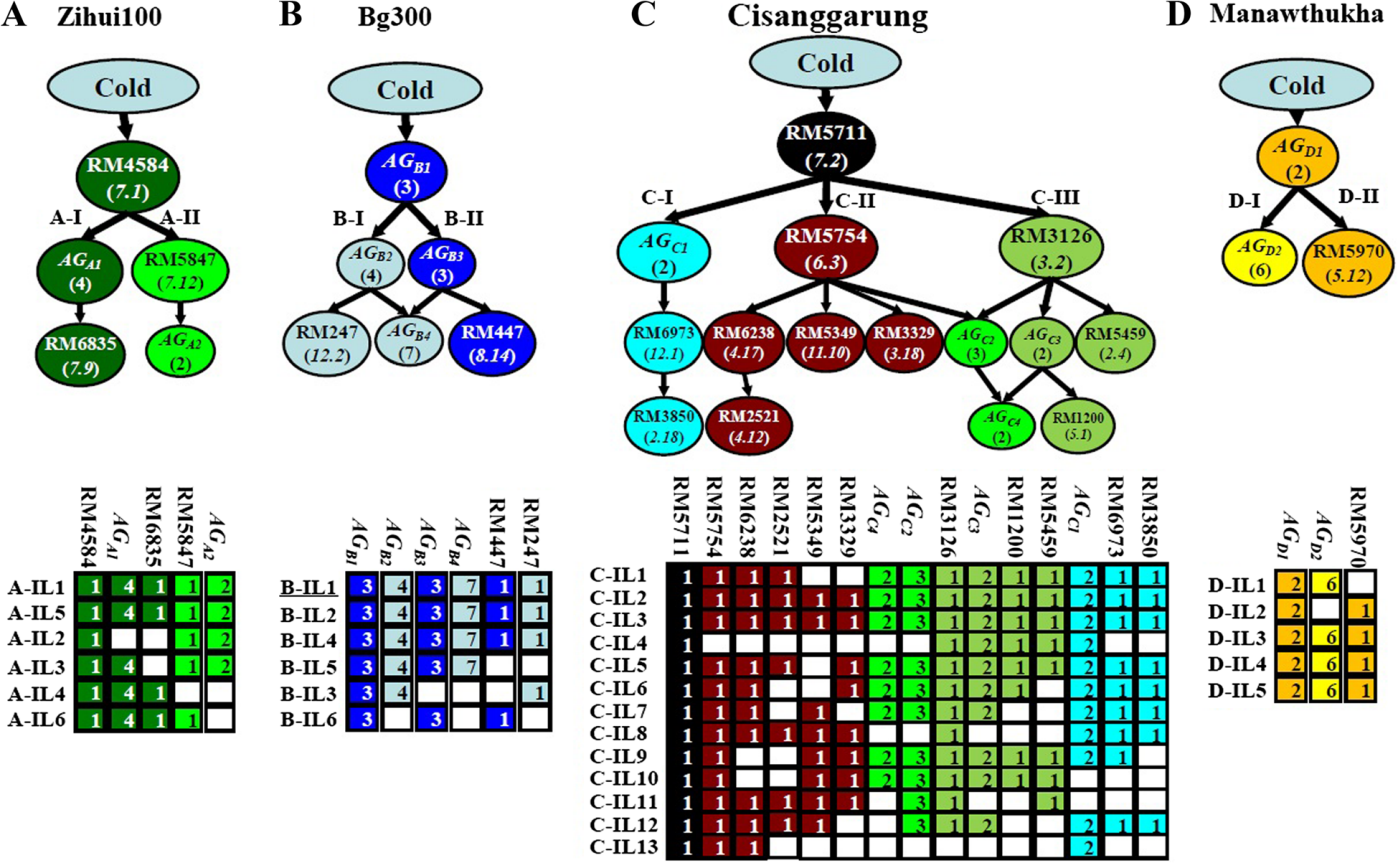
**Putative genetic networks underlying rice seedling cold tolerance (CT) detected in 30 cold-tolerant introgression lines (ILs) from four BC**_**2 **_**populations. (A)** Genetic network consisting of five functional genetic units (FGUs) and their graphic genotypes in six cold-tolerant ILs from the C418/Zihui100 population. **(B)** Genetic network consisting of six FGUs and their graphic genotypes in six cold-tolerant ILs from the C418/Bg300 population. **(C)** Genetic network comprising 15 FGUs detected in 13 cold-tolerant ILs from the C418/Cisanggarung population. **(D)** Genetic network consisting of three FGUs and their graphic genotypes in five cold-tolerant ILs from the C418/Manawthukha population. In graphical genotypes of each network, unfilled and fully colored cells represent the recipient homozygote and donor functional genotypes (donor homozygote plus the heterozygote), respectively. Numbers in cells of graphic genotypes are the number of loci included in the FGU (1 represents a single locus, while ≥ 2 represents an association group, AG). Here, an AG represents a group of unlinked but perfectly associated loci as shown in Table 3.

Of the 108 possible pairwise gametic-phase LD statistic (D*'*) values calculated between detected FGUs in cold-tolerant ILs from each BC population (Additional file 1: Table S3), 31 (28.7%) were statistically significant (Additional file 1: Table S4). The presence of significant non-random associations between or among alleles at unlinked CT loci in ILs possessing extreme (CT) phenotypes implied strong epistasis between or among these alleles. The results shown in Table S4 therefore allowed us to construct putative genetic networks underlying rice CT based on the principle of hierarchy [26] (Figure 2A–D).

Figure 2A is a representation of a putative genetic network containing five FGUs detected in the six cold-tolerant ILs of the C418/ZH100 population. The donor allele at RM4584 (bin 7.1) was present with an IF of 0.917 in all six cold-tolerant ILs, and was thus placed at the top of the network as the putative regulator. Two highly associated FGUs, *AG*_*A1*_ (bins 1.11, 1.15, 4.12, and 9.6) and RM6835 (bin 7.9) had lower IFs and formed the downstream branch A-I, while two highly associated FGUs, RM5847 (bin 7.12) and *AG*_*A2*_ (bins 6.3 and 6.15) formed the other downstream branch, A-II. FGUs in A-I and those in A-II were independent of one another (Additional file 1: Table S3). The six cold-tolerant ILs from this population could be classified into three major types based on their graphical genotypes at the five FGUs. IL1 and IL5 possessed donor alleles at both A-I and A-II. IL2 and IL3 had donor alleles at branch A-II, and IL4 featured donor alleles at branch A-I (Figure 2A). Interestingly, these ILs did not differ significantly from one another with respect to CT traits (Table 2), suggesting the functional redundancy of A-I and A-II FGUs with regard to their associations with CT traits.

Figure 2B illustrates the putative genetic network containing all six FGUs (four AGs and two loci) detected in the six ILs of the C418/Bg300 population. All six ILs were characterized by donor alleles at *AG*_*B1*_ (bins 2.6, 5.10, and 12.5) with IF = 0.833, which was thus placed at the top of the network as the putative regulator. Five unlinked but highly associated loci in two FGUs, *AG*_*B2*_ (bins 2.4, 2.11, 7.10, and 8.2) and RM247 (bin 12.2), had lower IFs, and formed downstream branch B-I. Similarly, branch B-II consisted of four unlinked but highly associated loci in two FGUs, *AG*_*B3*_ (bins 3.5, 6.3, and 9.9) and RM447 (bin 8.14), which had lower IFs and were thus placed downstream of *AG*_*B1*_. FGU *AG*_*B4*_ comprised seven unlinked but highly associated loci in bins 1.5, 3.12, 5.15, 6.12, 10.3, 10.9, and 11.14 (Table 3) that could be placed downstream of either branch B-I or B-II based on its significant associations with *AG*_*B2*_ or *AG*_*B3*_. The six ILs of this population were classifiable into four types. IL1, IL2, and IL4 had donor alleles at all loci of the six FGUs. IL5 had donor alleles at all loci except for the two single downstream loci (RM247 and RM447). IL3 featured donor alleles at branch B-I FGUs, while IL6 carried donor alleles at branch B-II FGUs. Again, phenotypic values of the CT-related traits did not seem to correlate with the number or type of downstream FGUs in the cold-tolerant ILs (Table 2).

Figure 2C depicts the putative genetic network comprising 15 FGUs (20 loci) detected in the 13 cold-tolerant ILs from the C418/CG population. RM5711 (bin 7.2) was placed at the top of the network as the putative regulator because all 13 cold-tolerant ILs had donor alleles at this locus. This network consisted of three major branches. *AG*_*C1*_ (bins 6.15 and 10.9) had very high IFs in the 13 ILs and thus was placed in the upper layer of branch C-I, which was connected with two associated loci located downstream at RM6973 (bin 12.1) and RM3850 (bin 2.18) with slightly lower IFs. Branch C-II had five FGUs, with RM5754 (bin 6.3) having the highest IF in the upper layer and significantly associated with four loci at bins 4.17, 11.10, 3.18, and 4.12 of lower IFs in three downstream sub-branches. Branch C-III was characterized by four FGUs, with RM3126 (bin 3.2) having the highest IF in the upper layer and associated with two downstream FGUs with lower IFs, *AG*_*C3*_ (bins 7.8 and 8.2) and RM5459 (bin 2.4). *AG*_*C2*_ (bins 3.8, 3.16, and 9.1) was significantly associated with RM5754 and RM3126, and thus could be placed downstream of either branch C-II or C-III; a similar situation existed with respect to *AG*_*C4*_ (bins 2.12 and 11.4). The 13 ILs from this population had different allelic combinations at loci in downstream FGUs in the network (Figure 2C), but most ILs, except for IL1, IL2, and IL3, were similar with regard to measured CT-related traits.

Figure 2D corresponds to the genetic network comprising three highly associated FGUs detected in the five cold-tolerant ILs from the C418/MNTH population. All five ILs of this population had donor alleles at two perfectly associated loci at bins 4.3 and 9.9 in *AG*_*D1*_; this AG was thus placed at the top of the network as the putative regulator. *AG*_*D1*_ was associated with seven unlinked loci in two other FGUs of lower IFs, including six highly associated downstream loci in *AG*_*D2*_ (bins 6.12, 7.5, 9.3, 10.10, 11.2, and 11.9) and RM5970 (bin 5.12). Again, the five ILs of this population were very similar with respect to the measured CT traits (Table 2), but they differed greatly in their allelic combinations at the two downstream FGUs.

## Discussion

With respect to the genetic basis of complex traits, selection and transgressive segregation have long been central issues in plant breeding and evolution. In this study, we developed 30 ILs that had significantly improved CT compared with their recurrent parent, C418. Our results indicate that all four *indica* donors contributed CT-enhancing alleles to the *japonica* recipient, C418. These results additionally imply the existence of a rich, hidden genetic diversity in the *indica* gene pool for improving *japonica* rice CT, as none of the donors were able to survive the same low temperature stress as the tested progeny in a preliminary experiment (unpublished observations). This type of transgressive segregation was observed for almost all complex traits in ~90% of the BC breeding populations in our large introgression breeding programs [20-24]. This phenomenon was our primary reason for not selecting parental lines based on their phenotype in any single target traits, as we were aiming to improve multiple complex traits using donors of diverse origins. Thus, our strategy of using introgression breeding, strong phenotypic selection plus genetic tracking and characterization of donor introgression using DNA markers has three main advantages that have been described previously [25]. These advantages are: (1) simultaneous improvement and genetic dissection of target traits under selection, (2) discovery and cross confirmation of a greater number of loci and alleles for the target trait from different donors, and (3) greater time savings and cost-effectiveness due to the small numbers of ILs of extreme phenotypes used for genotyping and phenotyping. Our results revealed two important aspects of genome-wide responses to strong phenotypic selection for CT in rice. The uncovered evidence may provide insights into the basis of the hidden genetic diversity and transgressive segregation of complex traits commonly observed in plant and animal breeding populations.

The first revealed aspect was the expected genome-wide significant allelic frequency shift, or over-introgression, of donor alleles at many loci across the genome in the selected cold-tolerant ILs. In this study, we detected 57 CT loci having an average IF of 0.469, or 3.75 times higher than expected (Table 3). Furthermore, 13 (~32%) of the 57 identified CT loci were simultaneously identified across multiple C418 BC populations having different *indica* donors (Figure 1). Fifteen (~37%) additional loci were verified in the segregating BC_2_F_2:6_ ILs from the originally selected BC_2_F_2_ plants (Table 3), and 29 (71%) loci were mapped to genomic regions previously reported to harbor major CT QTLs in different rice mapping populations (Figure 1; Table 3). Thus, few, if any, of the identified CT loci were false positives. The high efficiency in detecting CT loci using this selective introgression method is unsurprising, as the 30 cold-tolerant ILs were originally selected from 3,200 individuals in the four BC populations. This result meets the theoretical expectation that the stronger the selection intensity, the greater the amount of target-trait genetic information possessed by the selected progeny [29].

The second and most important aspect of genome-wide responses concerns the pronounced non-random associations observed between or among alleles at the detected CT loci, as reflected by the identification of 12 AGs and large numbers of significant LDs between different FGUs in the cold-tolerant ILs of each population. Historically, pronounced non-random associations between or among unlinked isozyme loci have been reported in self-pollinated plant species such as barley and rice, and have been interpreted as the consequence of selection operating on co-adapted gene complexes [30,31]. Unfortunately, genome-wide characterization of non-random associations between or among unlinked loci due to strong directional selection has rarely been carried out in experimental populations. Statistically, strong epistasis is implicated by strong non-random associations between or among alleles at unlinked loci in individuals of extreme phenotypes [26,30]. Indeed, six epistatic QTL pairs conferring CT (bin 3.5-bin 6.3, bin 6.3-bin 9.10, bin 4.12-bin 4.17, bin 4.17-bin 10.10, bin 6.13-bin 9.10, and bin 7.2-bin 11.10) that were previously reported in different rice mapping populations [12,13,16] were identified either as AGs or associated FGUs within the same branches of the CT genetic networks in this study (Figure 2; Additional file 1: Table S4). The presence of these loci in the same AG or in highly associated FGUs within the same branches of the putative genetic networks indicates that the donor alleles at the associated loci were co-responding to the strong selection for CT. These loci were therefore most likely involved in the same CT pathway or in related ones. We realize our strategy has several limitations. First, the small number of selected ILs had low power to detect significant loci and to differentiate IFs among significant FGUs and their hierarchical relationships. Second, owing to possible genetic drift, some of the significant LDs between and among unlinked FGUs may not represent true epistasis. Third, the pairwise LD analyses used in this study had low power to detect multi-locus structures with low and moderate IF when *r* was greater than 3. A more appropriate multi-locus probability test should take into consideration IF, the number of loci involved, and their non-random associations. Despite these limitations, we note two interesting properties of these putative genetic networks underlying rice CT, which resemble the gene networks of complex signaling pathways controlling abiotic stress tolerances in plants [32] and which have important biological implications.

The first property of interest involves the identification of some important genes controlling rice CT in bins 7.1–7.2, 2.6, 5.10, 12.5 (*AG*_*B1*_), 4.3, 9.9 (*AG*_*D1*_), 6.15, 10.9 (*AG*_*D1*_), 3.2, and 6.3. These loci were inferred as putative regulatory genes controlling rice CT pathways based on the theoretical expectation that regulatory genes respond to selection more strongly than do downstream ones [26] and the presence of donor alleles at these loci in all cold-tolerant ILs from each population (Figure 2). A bioinformatics search of these genomic regions revealed some interesting candidate genes. One of the three loci of *AG*_*B1*_ in bin 12.5 at the top of the C418/Bg300 CT-genetic network (Figure 2B) has been previously identified as a major CT QTL (*qCTS12*) and fine-mapped to the short arm of chromosome 12 [1,9] (Figure 1). Transcriptomic analyses of a cold-tolerant progeny, K354 derived from B-IL1 (Figure 2B) of the C418/Bg300 population, identified two regulatory genes in bin 12.5 that were highly up-regulated by cold: Sir2 protein (LOC_Os12g07950) and protein phosphatase 2C [31] genes. The former gene is related to epigenetic function, while the latter is involved in stress signaling as inferred by Gene Ontology analysis. Bin 2.6 harbors several possible candidate regulatory genes encoding proteins highly up-regulated by cold in K354: cytochrome P450s, terpene synthase (LOC_Os02g36140 and LOC_Os08g07100), NB-ARC domain-containing protein, histone deacetylase, and transcription factor *OsWRKY42*. Similarly, a large-effect CT QTL, *qCTS4*, has been identified and fine-mapped to bin 4.3 (*AG*_*D1*_) [1,18]. Bin 6.3 (Figure 2C) harbors a regulatory gene, *OsDREB1D*, that functions as one of several DREB transcription factors to enhance cold and salt tolerance in transgenic Arabidopsis [33] (Additional file 1: Table S5). Furthermore, bins 3.5 and 9.9 of *AG*_*B3*_ in the upper layer of the C418/Bg300 population network harbors four regulatory candidates, the signal transducer *OsMAPK5* and three transcription factors (*OsiSAP1*, *OsDREB1A* and *OsDREB1B*), all of which are known to play regulatory roles in rice CT [34-36] (Additional file 1: Table S5). The action of *AG*_*B3*_ (bins 3.5, 6.3, and 9.9) as a single FGU is also supported by the strong interactions among these CT QTLs in the same regions in another rice population [12]. Combined results from genetic and transcriptomic analyses thus appear to provide evidence for a role for these putative regulatory genes in control of rice CT. Nevertheless, over-expression and/or knockout experiments in transgenic plants are required to confirm whether any of these genes actually correspond to the important FGUs identified in the putative rice CT networks.

The second noted property is the fact that every putative genetic network consisted of two to three independent branches, each presumably corresponding to a putative downstream pathway as inferred from epistatic relationships. In addition, the phenotypic similarity in CT-related traits of ILs with different multilocus genotypes at these loci suggests the ‘functional redundancy’ of these rice CT downstream pathways. An extensive bioinformatics search of the Rice Genome Annotation Project database for genomic regions containing putative downstream CT FGUs identified several interesting positional candidate genes for rice CT. The identified candidates included six genes encoding putative cold-responsive (COR) proteins (COR_1–COR_5 and COR_7) located near three downstream FGUs adjacent to bin 2.18, *AG*_*C2*_ (bins 3.16 and 3.8), and *AG*_*B4*_ (bins 1.5, 5.15, and 6.12). Interestingly, the six downstream loci are all regulated by the rice DREB homolog *OsDREB1D* harbored in bin 6.3 [37], which was predicted to be the upstream locus of the six downstream loci in the putative CT genetic networks. In addition, genomic regions of *AG*_*B4*_ (bins 1.5, 5.15, and 6.12) in the C418/Bg300 population and two downstream loci near bins 5.12 and 6.12 in the C418/MNTH population harbor three COR genes (COR_3, COR_4, and COR_7) and one CT-related transcription factor (*AP37*), which are predicted to be regulated by one CT-related transcription factor, *OsiSAP1*, and two DREB homologs (*OsDREB1A* and *OsDREB1B*) near bin 9.9. Most of the above-mentioned CT candidate genes were found to be strongly up-regulated by cold in K354 derived from B-IL1 [38]. While individual characterization of the molecular functions of detected CT loci and putative pathways using the cold-tolerant ILs would be very challenging, genetic confirmation of putative CT genetic networks is not difficult. A straightforward strategy is to break each identified network or AG into individual loci and to evaluate their effects individually in progeny derived from crosses between selected ILs or between selected ILs and recurrent parent C418.

Evolutionally, the above-described genetic system underlying complex traits is expected maintain high levels of genetic variation even in populations under strong directional selection. For example, although all 30 cold-tolerant ILs selected in this study reached fixation after two rounds of selection for CT followed by two additional generations of selfing, significant amounts of genetic variation existed at all downstream loci in cold-tolerant ILs from each population. The same situation was observed for putative upstream regulatory loci when ILs from different populations were considered. This type of genetic system, with multiple pathways affecting the same traits, obviously can be (and indeed may have been) responsible for the maintenance of the tremendous genetic variation at loci for complex traits in plant and animal species, even under long-term directional selection [39,40].

Our results have important implications for improving complex traits in rice. Our data indicate that genetic complementarity and repulsive distribution of functional alleles in the parents provides an appropriate explanation for the hidden diversity and transgressive segregation of CT, observed in this study, and other complex traits in rice [20-24]. Examination of graphic genotypes of the cold-tolerant ILs (Figure 2A–D) revealed that each IL had the functional (donor) alleles of at least one upstream locus plus one or more downstream FGUs. This observation implies that the improved CT of the C418 ILs was apparently achieved by complementation of one or more broken pathways from introgressed *indica* alleles. The *japonica* rice gene pool is known to have very limited genetic diversity [5,31]. In addition, past rice breeding of both *indica* and *japonica* rice has focused on exploitation of within-subspecies diversity, as hybrid sterility and hybrid breakdown are present in populations derived from inter-subspecific crosses [41]. This general tendency of narrowing the genetic basis of the breeding parents followed by continued selection for the same suite of target traits in specific breeding populations would be expected to result in the fixation of functional alleles, with fewer pathways affecting target traits in progeny selected from the same population and, conversely, more differentiation observed in progeny selected from different populations (Figure 2). Tremendous efforts should therefore be taken to broaden the genetic diversity of the elite rice gene pool. This goal may be accomplished by exploiting hidden diversity from distantly related parents or exotic germplasm, such as landraces and accessions of different subspecies, which are more likely to have complementary functional alleles for the broken pathways affecting complex traits in elite rice varieties [42].

## Conclusions

Strong phenotypic selection for rice seedling CT in four rice BC populations resulted in the development of 30 cold-tolerant ILs. Genetic tracking and characterization of donor introgression in the 30 selected ILs using DNA markers revealed two important aspects of genome-wide responses to selection: (1) significant over-introgression of donor alleles at 57 loci across the rice genome, and (2) pronounced non-random associations resulting from strong epistasis between or among many alleles at unlinked loci. LD analyses of identified CT loci allowed us to infer putative genetic networks or multi-locus structures underlying rice seedling CT. Our results indicate that genetic complementarity and repulsive distribution of functional alleles in the parents provides an appropriate explanation for the hidden diversity and transgressive segregation of CT observed in this study and other complex traits in rice. Our results suggest that complex traits such as CT in rice are controlled by multiple pathways, each involving large numbers of loci, which are able to maintain high levels of genetic variation at loci affecting complex traits even under long-term directional selection.

## Abbreviations

FGU: Functional genetic unit; IL: Introgression line; CT: Cold tolerance; RP: Recurrent parent; SDW: Seedling dry weight; CRI: Cold response index; SP: Seedling survival percentage; SSR: Simple sequence repeat; ZH100: Zihui100; Cis: Cisanggarung; MNTH: Manawthukha; FD: Functional dependency; IF: Introgression frequency; AG: Association group; LD: Linkage disequilibrium.

## Competing interests

The authors declare that they have no competing interests.

## Authors’ contributions

LZK conceived and designed the research, and helped to draft the manuscript. GYM, MXF and ZF performed the statistical analysis. HXB, MXF and ZF collected the phenotyping data and participated in the molecular genotyping. ZF interpreted the results and drafted the manuscript. All authors read and approved the final manuscript.

## Supplementary Material

Additional file 1: Table S1 The list of 298 polymorphic SSR markers in 137 bins across the rice genome used to genotype the introgression lines from the four BC populations. Additional file 1: Table S2. Detailed information on linkage disequilibrium (LD) analysis of the perfect association groups (AGs) for seedling cold tolerance detected in ILs selected from four BC_2_F_2_ populations between C418 and four different *indica* donors. Additional file 1: Table S3. Summarized results of 108 pairwise LD analyses between identified FGUs, either individual loci of excess introgression or perfect AGs, for seedling cold tolerance (CT) detected in 30 cold-tolerant ILs from four BC_2_F_2_ between C418 and four different donors. Additional file 1: Table S4. Previously reported CT epistatic QTL intervals consistent with the relationships among FGUs of putative CT genetic networks detected in this study. Additional file 1: Table S5. Signal transducers, transcription factors, and responsive proteins related to CT near or within regions of the FGUs detected in this study. Click here for file

Additional file 2: Figure S1 Seedling cold-tolerance phenotypes of introgression lines derived from four recurrent parent C418 and four BC_2_F_2_ populations with four different *indica* donors. Treatments consisted of exposure to 4°C for 7 d in a growth chamber (A) and natural low-temperature conditions (B and C). (A) Seedling survival percentage. (B) Cold tolerance index of seedling height. (C) Cold tolerance index of seedling dry weight. Click here for file
