# Supplementary material for: Genome-wide response to selection and genetic basis of cold tolerance in rice (Oryza sativa L.)
Source: BMC Genet. 2014 May 8;15:55. doi: 10.1186/1471-2156-15-55 (PMC4024214; doi:10.1186/1471-2156-15-55)
Supplement: Additional file 1: Table S1 — The list of 298 polymorphic SSR markers in 137 bins across the rice genome used to genotype the introgression lines from the four BC populations. Additional file 1: Table S2. Detailed information on linkage disequilibrium (LD) analysis of the perfect association groups (AGs) for seedling cold tolerance detected in ILs selected from four BC2F2 populations between C418 and four different indica donors. Additional file 1: Table S3. Summarized results of 108 pairwise LD analyses between identified FGUs, either individual loci of excess introgression or perfect AGs, for seedling cold tolerance (CT) detected in 30 cold-tolerant ILs from four BC2F2 between C418 and four different donors. Additional file 1: Table S4. Previously reported CT epistatic QTL intervals consistent with the relationships among FGUs of putative CT genetic networks detected in this study. Additional file 1: Table S5. Signal transducers, transcription factors, and responsive proteins related to CT near or within regions of the FGUs detected in this study. [file 1471-2156-15-55-S1.doc]

**Genome-wide Response to Selection and Genetic Basis of Cold Tolerance in Rice (*Oryza sativa* L.)**

Fan Zhang^1*^, Xiu-Fang Ma^2*^, Yong-Ming Gao^1^, Xian-Bin Hao^2^, Zhi-Kang Li ^1§^

^1^Institute of Crop Sciences/National Key Facility for Crop Gene Resources and Genetic Improvement, Chinese Academy of Agricultural Sciences, Beijing, China

^2^Liaoning Academy of Agricultural Sciences, Shenyang, Liaoning, China

*These authors contributed equally to this work

^§^Corresponding author

Email addresses:

FZ: [zhangfan03@caas.cn](mailto:zhangfan03@caas.cn)

XFM: [mellowmxf@163.com](mailto:mellowmxf@163.com)

YMG: [irriygao@126.com](mailto:irriygao@126.com)

XBH: [xianbinhao2014@126.com](mailto:xianbinhao2014@126.com)

ZKL: [lizhikang@caas.cn](mailto:lizhikang@caas.cn)

**Additional file 1**

**Table S1** The list of 298 polymorphic SSR markers in 137 bins across the rice genome used to genotype the introgression lines from the four BC populations

| Marker | Chr. | Genetic distance (cM)^1^ | Physical position  (bp)^1^ | Bin ID^2^ | Polymophic SSR markers used for genotyping in different populations^3^ | | | |
| --- | --- | --- | --- | --- | --- | --- | --- | --- |
|  |  |  |  |  | ZH100 | Bg300 | Cis | MNTH |
| RM4554 | 1 | 0 | 388932-388975 | Bin1.1 | . | . | √ | . |
| RM1247 | 1 | 9.5 | 1658105-1658134 | Bin1.1 | . | . | √ | √ |
| RM1331 | 1 | 9.5 | 1669200-1669239 | Bin1.1 | . | . | √ | . |
| RM6045 | 1 | 10.9 | 1775853-1775879 | Bin1.1 | . | . | . | √ |
| RM7278 | 1 | 10.9 | 1793925-1793964 | Bin1.1 | √ | . | . | . |
| RM5336 | 1 | 16.1 | 3366409-3366432 | Bin1.2 | . | . | . | √ |
| RM490 | 1 | 51 | 6676195-6676318 | Bin1.4 | . | √ | . | . |
| RM6786 | 1 | 32.4 | 7075365-7075385 | Bin1.4 | √ | . | . | . |
| RM8098 | 1 | 36.9 | 7246143-7246194 | Bin1.4 | √ | . | . | . |
| RM5496 | 1 | 42.2 | 8074257-8074304 | Bin1.5 | . | . | . | √ |
| RM579 | 1 | 61.3 | 8451657-8451706 | Bin1.5 | . | √ | . | . |
| RM5346 | 1 | 45.4 | 8750110-8750135 | Bin1.5 | . | . | √ | . |
| RM8067 | 1 | 50.8 | 9605723-9605779 | Bin1.5 | . | . | √ | √ |
| RM8051 | 1 | 52.1 | 9865594-9865621 | Bin1.5 | √ | . | . | . |
| RM449 | 1 | 82 | 15106279-15106302 | Bin1.8 | . | √ | . | . |
| RM6711 | 1 | 73.4 | 16094779-16094802 | Bin1.9 | . | . | . | √ |
| RM129 | 1 | 78.4 | 19005894-19005917 | Bin1.10 | . | √ | . | . |
| RM5638 | 1 | 86 | 20932038-20932076 | Bin1.11 | √ | . | . | . |
| RM3341 | 1 | 90 | 21883961-21883990 | Bin1.11 | √ | . | . | . |
| RM1196 | 1 | 87.4 | 21900509-21900536 | Bin1.11 | √ | . | . | √ |
| RM7124 | 1 | 101.8 | 24386986-24387021 | Bin1.13 | √ | . | √ | √ |
| RM488 | 1 | 101.4 | 24804715-24804748 | Bin1.13 | . | √ | . | . |
| RM3143 | 1 | 112.7 | 26814912-26814945 | Bin1.14 | √ | . | √ | . |
| RM246 | 1 | 115.2 | 27329504-27329539 | Bin1.14 | . | √ | . | . |
| RM443 | 1 | 122.7 | 28333781-28333800 | Bin1.15 | . | √ | . | . |
| RM1297 | 1 | 121.6 | 28606563-28606598 | Bin1.15 | √ | . | . | . |
| RM5914 | 1 | 129.3 | 31503646-31503717 | Bin1.16 | √ | . | . | √ |
| RM297 | 1 | 155.9 | 32093949-32094020 | Bin1.17 | . | √ | . | . |
| RM212 | 1 | 148.7 | 33047896-33047923 | Bin1.17 | . | √ | . | . |
| RM7594 | 1 | 141.6 | 35465505-35465528 | Bin1.18 | √ | . | √ | √ |
| RM8134 | 1 | 181.8 | 38888469-38888500 | Bin1.20 | . | . | √ | . |
| RM104 | 1 | 186.6 | 40166840-40167060 | Bin1.21 | . | √ | . | . |
| RM1387 | 1 | 159 | 40201060-40201147 | Bin1.21 | √ | . | . | . |
| RM5310 | 1 | 164.1 | 41190755-41190784 | Bin1.21 | √ | √ | . | √ |
| RM8137 | 1 | 181.8 | 42918106-42918129 | Bin1.22 | √ | . | . | √ |
| RM3340 | 2 | 0 | 386212-386241 | Bin2.1 | √ | . | √ | . |
| RM236 | 2 | 14.4 | 2105660-2105691 | Bin2.2 | . | √ | . | . |
| RM4355 | 2 | 20.9 | 4264318-4264355 | Bin2.3 | √ | . | . | . |
| RM7215 | 2 | 25.5 | 4790692-4790731 | Bin2.3 | . | . | . | √ |
| RM5897 | 2 | 32.8 | 6732689-6732733 | Bin2.4 | √ | . | . | √ |
| RM5459 | 2 | 36.3 | 7518236-7518271 | Bin2.4 | . | . | √ | . |
| RM145 | 2 | 49.8 | 7706071-7706132 | Bin2.4 | . | √ | . | . |
| RM1234 | 2 | 51.1 | 11335517-11335546 | Bin2.6 | √ | . | . | . |
| RM324 | 2 | 66 | 11388913-11388939 | Bin2.6 | . | √ | . | . |
| RM1211 | 2 | 62.2 | 18556118-18556145 | Bin2.10 | . | . | . | √ |
| RM3858 | 2 | 62.2 | 19208210-19208261 | Bin2.10 | . | . | √ | . |
| RM2634 | 2 | 81 | 20520574-20520593 | Bin2.11 | . | . | √ | . |
| RM262 | 2 | 82.7 | 20794972-20795112 | Bin2.11 | . | √ | . | . |
| RM3688 | 2 | 88.2 | 22420221-22420250 | Bin2.12 | √ | . | √ | √ |
| RM3355 | 2 | 95.2 | 24040757-24040786 | Bin2.13 | . | √ | . | . |
| RM3515 | 2 | 95.5 | 24041889-24041944 | Bin2.13 | . | . | . | √ |
| RM263 | 2 | 127.5 | 25889828-25889855 | Bin2.13 | . | √ | . | . |
| RM1367 | 2 | 110.6 | 27083859-27083912 | Bin2.14 | √ | . | √ | . |
| RM221 | 2 | 143.7 | 27609877-27610063 | Bin2.14 | . | √ | . | . |
| RM6933 | 2 | 123.9 | 29331976-29332077 | Bin2.15 | √ | . | √ | √ |
| RM318 | 2 | 150.8 | 29655977-29655998 | Bin2.15 | . | √ | . | . |
| RM3316 | 2 | 130.2 | 30603641-30603668 | Bin2.16 | . | . | √ | . |
| RM240 | 2 | 158 | 31497147-31497256 | Bin2.16 | . | √ | . | . |
| RM112 | 2 | 166 | 32013785-32013912 | Bin2.17 | . | √ | . | . |
| RM7286 | 2 | 141.7 | 33019042-33019105 | Bin2.17 | √ | . | . | √ |
| RM3850 | 2 | 157.9 | 35450187-35450234 | Bin2.18 | √ | . | √ | √ |
| RM4108 | 3 | 1.1 | 514907-514934 | Bin3.1 | √ | . | . | . |
| RM523 | 3 | 11.1 | 1300317-1300344 | Bin3.1 | . | √ | . | . |
| RM3372 | 3 | 6 | 1437206-1437237 | Bin3.1 | . | . | . | √ |
| RM3126 | 3 | 16.8 | 3661277-3661304 | Bin3.2 | √ | . | √ | √ |
| RM5474 | 3 | 17.9 | 3785967-3786008 | Bin3.2 | √ | . | √ | . |
| RM3392 | 3 | 18.7 | 3808795-3808828 | Bin3.2 | . | . | . | √ |
| RM175 | 3 | 19.5 | 3848611-3848634 | Bin3.2 | . | √ | . | . |
| RM4352 | 3 | 20.3 | 4296987-4297020 | Bin3.3 | √ | . | √ | . |
| RM489 | 3 | 29.2 | 4316616-4316639 | Bin3.3 | . | √ | . | . |
| RM2790 | 3 | 21.5 | 4505458-4505491 | Bin3.3 | . | . | √ | √ |
| RM1278 | 3 | 21.5 | 4544321-4544354 | Bin3.3 | √ | . | . | √ |
| RM2791 | 3 | 157.7 | 4552353-4552420 | Bin3.3 | √ | . | . | . |
| RM6038 | 3 | 24.4 | 4811234-4811260 | Bin3.3 | . | . | . | √ |
| RM6472 | 3 | 99 | 5364840-5364866 | Bin3.3 | . | . | . | √ |
| RM5393 | 3 | 146.1 | 5507754-5507773 | Bin3.3 | . | . | √ | . |
| RM6058 | 3 | 31.1 | 6528967-6528990 | Bin3.4 | √ | . | . | . |
| RM5896 | 3 | 40.3 | 8515358-8515399 | Bin3.5 | . | . | . | √ |
| RM7 | 3 | 64 | 9828245-9828447 | Bin3.5 | . | √ | . | . |
| RM251 | 3 | 79.1 | 9948792-9948961 | Bin3.5 | . | √ | . | . |
| RM3803 | 3 | 48.5 | 10628316-10628353 | Bin3.6 | √ | . | . | . |
| RM7365 | 3 | 49.3 | 11249238-11249259 | Bin3.6 | √ | √ | . | . |
| RM5748 | 3 | 55.8 | 12297768-12297803 | Bin3.7 | . | . | √ | . |
| RM3257 | 3 | 61.9 | 13243919-13243944 | Bin3.7 | . | . | √ | . |
| RM6959 | 3 | 65.4 | 14341066-14341092 | Bin3.8 | √ | . | √ | . |
| RM3400 | 3 | 84.4 | 17072066-17072099 | Bin3.9 | . | . | . | √ |
| RM1334 | 3 | 84.4 | 17228486-17228527 | Bin3.9 | √ | . | √ | . |
| RM156 | 3 | 85.8 | 17714639-17714821 | Bin3.9 | . | √ | . | . |
| RM5488 | 3 | 87.1 | 21193831-21193876 | Bin3.11 | √ | . | . | . |
| RM3698 | 3 | 87.9 | 21585837-21585866 | Bin3.11 | . | . | . | √ |
| RM6832 | 3 | 89 | 22203339-22203362 | Bin3.12 | . | √ | . | . |
| RM2334 | 3 | 115.6 | 26547397-26547446 | Bin3.14 | √ | . | √ | . |
| RM1350 | 3 | 126.8 | 28479832-28479877 | Bin3.15 | √ | . | . | . |
| RM186 | 3 | 168.2 | 28806696-28806820 | Bin3.15 | . | √ | . | . |
| RM183 | 3 | . | 29306061-29306305 | Bin3.15 | . | √ | . | . |
| RM3199 | 3 | 137.6 | 30221208-30221231 | Bin3.16 | √ | . | √ | √ |
| RM3329 | 3 | 158.2 | 35381850-35381879 | Bin3.18 | . | . | √ | . |
| RM148 | 3 | 218.5 | 35835805-35836096 | Bin3.18 | . | √ | . | . |
| RM85 | 3 | 231 | 36341096-36341225 | Bin3.18 | . | √ | . | . |
| RM1867 | 4 | 5.4 | 922396-922605 | Bin4.1 | √ | . | . | . |
| RM6487 | 4 | 12.2 | 4647002-4647025 | Bin4.3 | . | √ | . | . |
| RM3658 | 4 | 14.3 | 5573675-5573702 | Bin4.3 | . | . | . | √ |
| RM5633 | 4 | 20.9 | 13135281-13135316 | Bin4.7 | . | . | √ | √ |
| RM307 | 4 | 0 | 13141577-13141773 | Bin4.7 | . | √ | . | . |
| RM6314 | 4 | 41.5 | 18627879-18627911 | Bin4.10 | . | √ | . | . |
| RM3308 | 4 | 44 | 19186623-19186650 | Bin4.10 | . | . | √ | . |
| RM3742 | 4 | 52.6 | 19925263-19925296 | Bin4.10 | . | . | . | √ |
| RM1359 | 4 | 56.1 | 20041155-20041204 | Bin4.11 | √ | . | . | √ |
| RM1155 | 4 | 58.9 | 20525179-20525204 | Bin4.11 | . | √ | . | . |
| RM5979 | 4 | 60.2 | 20780840-20780884 | Bin4.11 | . | . | √ | √ |
| RM2521 | 4 | 70.1 | 23193002-23193075 | Bin4.12 | √ | . | √ | . |
| RM3866 | 4 | 23.3 | 23333714-23333769 | Bin4.12 | √ | . | . | . |
| RM3839 | 4 | 72.8 | 24065173-24065218 | Bin4.13 | √ | . | √ | √ |
| RM252 | 4 | 99 | 25179120-25179358 | Bin4.13 | . | √ | . | . |
| RM1223 | 4 | 78.2 | 25451428-25451457 | Bin4.13 | √ | . | . | . |
| RM1354 | 4 | 78.2 | 26272526-26272571 | Bin4.14 | √ | . | √ | . |
| RM3916 | 4 | 94.4 | 28733281-28733304 | Bin4.15 | √ | . | √ | √ |
| RM317 | 4 | 118.3 | 29219406-29219425 | Bin4.15 | . | √ | . | . |
| RM6480 | 4 | 99.3 | 29852235-29852261 | Bin4.15 | √ | . | √ | . |
| RM3534 | 4 | 107.4 | 31198068-31198091 | Bin4.16 | √ | . | √ | √ |
| RM255 | 4 | 109.4 | 30991473-30991510 | Bin4.16 | . | √ | . | . |
| RM6238 | 4 | 113.2 | 33040361-33040387 | Bin4.17 | . | . | √ | . |
| RM348 | 4 | 136.2 | 32869438-32869458 | Bin4.17 | . | √ | . | . |
| RM2431 | 4 | 129.6 | 35139856-35139883 | Bin4.18 | . | . | √ | √ |
| RM280 | 4 | 152.3 | 35209912-35209933 | Bin4.18 | . | √ | . | . |
| RM1248 | 5 | 0.3 | 62666-62695 | Bin5.1 | √ | . | √ | √ |
| RM1200 | 5 | 9.5 | 804991-805018 | Bin5.1 | √ | . | √ | √ |
| RM1024 | 5 | 12.5 | 1174375-1174400 | Bin5.1 | . | √ | . | . |
| RM6517 | 5 | 24.7 | 2853314-2853337 | Bin5.2 | √ | . | √ | √ |
| RM267 | 5 | 31.4 | 2821024-2821047 | Bin5.2 | . | √ | . | . |
| RM7118 | 5 | 41.4 | 6005524-6005551 | Bin5.4 | √ | . | . | . |
| RM13 | 5 | 28.6 | 11606802-11606965 | Bin5.6 | . | √ | . | . |
| RM6742 | 5 | 55.4 | 14743687-14743752 | Bin5.8 | √ | . | . | √ |
| RM164 | 5 | 78.7 | 19114842-19114873 | Bin5.10 | . | √ | . | . |
| RM1386 | 5 | 75 | 19912953-19913036 | Bin5.10 | √ | . | . | . |
| RM3800 | 5 | 87.4 | 21309979-21310002 | Bin5.11 | √ | . | . | . |
| RM173 | 5 | 99.8 | 21644432-21644617 | Bin5.11 | . | √ | . | . |
| RM3295 | 5 | 94.5 | 22187443-22187470 | Bin5.12 | √ | . | √ | √ |
| RM5311 | 5 | 95.8 | 22828546-22828569 | Bin5.12 | . | . | . | √ |
| RM3476 | 5 | 101 | 23761421-23761464 | Bin5.12 | . | . | √ | √ |
| RM5970 | 5 | 101.5 | 23863707-23863730 | Bin5.12 | . | . | . | √ |
| RM3809 | 5 | 108.5 | 26444281-26444320 | Bin5.14 | . | . | . | √ |
| RM7452 | 5 | 110.4 | 26783037-26783072 | Bin5.14 | . | . | . | √ |
| RM274 | 5 | 126.6 | 26848154-26848303 | Bin5.14 | . | √ | . | . |
| RM2357 | 5 | 109 | 26712744-26712793 | Bin5.14 | . | . | . | √ |
| RM6360 | 5 | 115.7 | 27924665-27924697 | Bin5.14 | . | . | . | √ |
| RM31 | 5 | 118.8 | 28453105-28453144 | Bin5.15 | . | √ | . | . |
| RM334 | 5 | 141.8 | 28484755-28484959 | Bin5.15 | . | √ | . | . |
| RM6467 | 6 | 0.6 | 220065-220088 | Bin6.1 | √ | . | √ | √ |
| RM540 | 6 | 0 | 381287-381481 | Bin6.1 | . | √ | . | . |
| RM469 | 6 | 2.2 | 564234-564263 | Bin6.1 | . | √ | . | . |
| RM587 | 6 | 10.7 | 2291878-2291931 | Bin6.2 | . | √ | . | . |
| RM510 | 6 | 20.8 | 2831543-2831572 | Bin6.2 | . | √ | . | . |
| RM217 | 6 | 26.2 | 4234228-4234383 | Bin6.3 | . | √ | . | . |
| RM3408 | 6 | 15.8 | 4608700-4608733 | Bin6.3 | √ | . | √ | √ |
| RM5754 | 6 | 31.3 | 5245011-5245064 | Bin6.3 | √ | . | √ | √ |
| RM253 | 6 | 37 | 5437340-5437383 | Bin6.3 | . | √ | . | . |
| RM2615 | 6 | 32.7 | 5970899-5970960 | Bin6.3 | . | . | √ | √ |
| RM5855 | 6 | 40.2 | 7422774-7422860 | Bin6.4 | √ | . | √ | √ |
| RM136 | 6 | 51.2 | 8763322-8763342 | Bin6.5 | . | √ | . | . |
| RM5963 | 6 | 51 | 8826489-8826515 | Bin6.5 | √ | . | √ | . |
| RM527 | 6 | 61.2 | 9874150-9874183 | Bin6.5 | . | √ | . | . |
| RM3330 | 6 | 61.6 | 10907997-10908028 | Bin6.6 | √ | . | √ | . |
| RM7193 | 6 | 70.6 | 19910922-19910949 | Bin6.11 | √ | . | √ | √ |
| RM6298 | 6 | 85.4 | 23353565-23353588 | Bin6.12 | . | . | . | √ |
| RM5957 | 6 | 90.5 | 24138891-24138914 | Bin6.13 | . | . | √ | . |
| RM275 | 6 | 95 | 24324733-24324821 | Bin6.13 | . | √ | . | . |
| RM6782 | 6 | 99.2 | 25662672-25662701 | Bin6.13 | . | . | . | √ |
| RM7309 | 6 | 100.3 | 25914707-25914734 | Bin6.13 | . | . | . | √ |
| RM5988 | 6 | 105.1 | 26873633-26873656 | Bin6.14 | √ | . | . | . |
| RM3430 | 6 | 107.3 | 27050051-27050086 | Bin6.14 | . | . | . | √ |
| RM3 | 6 | 109.5 | 27252152-27252383 | Bin6.14 | . | √ | . | . |
| RM3307 | 6 | 113.4 | 28563720-28563747 | Bin6.15 | √ | . | √ | . |
| RM7555 | 6 | 117 | 29054909-29054956 | Bin6.15 | . | . | √ | . |
| RM5463 | 6 | 124.4 | 30470162-30470199 | Bin6.15 | √ | √ | . | . |
| RM295 | 7 | 0.8 | 413668-413854 | Bin7.1 | . | √ | . | . |
| RM4584 | 7 | 2.2 | 494948-494991 | Bin7.1 | √ | . | . | . |
| RM7454 | 7 | 4.4 | 1102352-1102379 | Bin7.1 | . | . | √ | . |
| RM3325 | 7 | 9.6 | 2250449-2250476 | Bin7.2 | . | . | √ | √ |
| RM5711 | 7 | 24.2 | 3174356-3174427 | Bin7.2 | √ | . | √ | . |
| RM6728 | 7 | 42.6 | 5762230-5762277 | Bin7.3 | . | . | √ | . |
| RM8006 | 7 | 46.5 | 7718306-7718335 | Bin7.4 | √ | . | √ | . |
| RM3718 | 7 | 47.7 | 7989385-7989416 | Bin7.4 | √ | . | . | . |
| RM3859 | 7 | 49.4 | 8910448-8910503 | Bin7.5 | √ | . | . | √ |
| RM5436 | 7 | 49.7 | 9107916-9107949 | Bin7.5 | √ | . | . | √ |
| RM3635 | 7 | 49.7 | 11120525-11120550 | Bin7.6 | √ | . | √ | . |
| RM3755 | 7 | 50.9 | 14680995-14681028 | Bin7.8 | √ | . | √ | . |
| RM5875 | 7 | 55.6 | 15945284-15945307 | Bin7.8 | √ | . | √ | . |
| RM5793 | 7 | 60.8 | 17437134-17437157 | Bin7.9 | √ | . | . | . |
| RM6835 | 7 | 60.8 | 17638644-17638682 | Bin7.9 | √ | . | . | . |
| RM432 | 7 | 43.5 | 18906053-18906088 | Bin7.10 | . | √ | . | . |
| RM3826 | 7 | 73.2 | 20754864-20754905 | Bin7.11 | √ | . | √ | . |
| RM6011 | 7 | 73.2 | 20733917-20733940 | Bin7.11 | . | √ | . | . |
| RM346 | 7 | 56.4 | 21045102-21045297 | Bin7.11 | . | √ | . | . |
| RM455 | 7 | 65.7 | 22298054-22298073 | Bin7.12 | . | √ | . | . |
| RM5847 | 7 | 80.5 | 23596026-23596100 | Bin7.12 | √ | . | √ | √ |
| RM118 | 7 | 96.9 | 26637598-26637752 | Bin7.14 | . | √ | . | . |
| RM1364 | 7 | 99.6 | 26721471-26721522 | Bin7.14 | √ | . | . | . |
| RM7601 | 7 | 116.6 | 28986007-28986034 | Bin7.15 | √ | . | √ | √ |
| RM248 | 7 | 116.6 | 29287459-29287488 | Bin7.15 | . | √ | . | . |
| RM6369 | 8 | 0 | 119337-119378 | Bin8.1 | √ | . | √ | . |
| RM337 | 8 | 1.1 | 152299-152485 | Bin8.1 | . | √ | . | . |
| RM1381 | 8 | 1.9 | 424202-424399 | Bin8.1 | . | . | √ | . |
| RM6356 | 8 | 13.4 | 1555490-1555519 | Bin8.1 | . | . | √ | . |
| RM38 | 8 | 28 | 2109541-2109576 | Bin8.2 | . | √ | . | . |
| RM1148 | 8 | 31.6 | 3733809-3733832 | Bin8.2 | √ | . | √ | √ |
| RM547 | 8 | 58.1 | 5586081-5586140 | Bin8.3 | . | √ | . | . |
| RM6838 | 8 | 42.9 | 5844242-5844283 | Bin8.3 | √ | . | . | √ |
| RM3395 | 8 | 52.9 | 10288496-10288529 | Bin8.6 | . | . | . | √ |
| RM331 | 8 | 69 | 12288130-12288159 | Bin8.7 | . | √ | . | . |
| RM5767 | 8 | 61.2 | 18814405-18814431 | Bin8.10 | √ | . | √ | √ |
| RM223 | 8 | 80.5 | 20650060-20650244 | Bin8.11 | . | √ | . | . |
| RM3634 | 8 | 82.8 | 21575892-21575917 | Bin8.11 | √ | . | √ | √ |
| RM6070 | 8 | 108.2 | 26315198-26315221 | Bin8.14 | . | . | . | √ |
| RM447 | 8 | 124.6 | 26542515-26542538 | Bin8.14 | . | √ | . | . |
| RM3480 | 8 | 118.9 | 27386021-27386064 | Bin8.14 | √ | . | √ | . |
| RM316 | 9 | 1.8 | 1022756-1022775 | Bin9.1 | . | √ | . | . |
| RM3609 | 9 | 0.8 | 1104566-1104591 | Bin9.1 | √ | . | √ | √ |
| RM5899 | 9 | 2.1 | 5070062-5070109 | Bin9.3 | . | . | √ | √ |
| RM6021 | 9 | 2.4 | 5254503-5254526 | Bin9.3 | √ | . | √ | . |
| RM7481 | 9 | 10 | 6540799-6540822 | Bin9.4 | . | . | . | √ |
| RM219 | 9 | 11.7 | 7888380-7888602 | Bin9.4 | . | √ | . | . |
| RM1328 | 9 | 26.7 | 9152293-9152332 | Bin9.5 | √ | . | √ | √ |
| RM5777 | 9 | 33 | 10075999-10076031 | Bin9.6 | . | . | √ | . |
| RM296 | 9 | 22.2 | 10785115-10785217 | Bin9.6 | . | √ | . | . |
| RM1896 | 9 | 36 | 11714708-11714727 | Bin9.6 | √ | . | √ | . |
| RM6235 | 9 | 60.8 | 16620811-16620834 | Bin9.9 | √ | . | √ | √ |
| RM257 | 9 | 66.1 | 17666088-17666147 | Bin9.9 | . | √ | . | . |
| RM6543 | 9 | 65.1 | 17696455-17696481 | Bin9.9 | . | . | . | √ |
| RM288 | 9 | 74.6 | 8562723-18562846 | Bin9.10 | . | √ | . | . |
| RM6570 | 9 | 68.2 | 18576133-18576251 | Bin9.10 | √ | . | √ | . |
| RM328 | 9 | 82.4 | 19725385-19725577 | Bin9.10 | . | √ | . | . |
| RM3808 | 9 | 78.8 | 20246518-20246557 | Bin9.11 | √ | . | . | . |
| RM1553 | 9 | 82.1 | 20702581-20702606 | Bin9.11 | √ | . | . | . |
| RM215 | 9 | 99.4 | 20888227-20888258 | Bin9.11 | . | √ | . | . |
| RM6971 | 9 | 90.4 | 21591824-21591862 | Bin9.11 | √ | . | √ | √ |
| RM245 | 9 | 112.3 | 22274644-22274814 | Bin9.11 | . | √ | . | . |
| RM6797 | 9 | 93.5 | 22412138-22412161 | Bin9.12 | √ | . | . | . |
| RM7492 | 10 | 0.6 | 33968-33995 | Bin10.1 | √ | . | √ | . |
| RM3882 | 10 | 5.5 | 2717526-2717549 | Bin10.2 | √ | . | √ | . |
| RM216 | 10 | 17.6 | 4986973-4986996 | Bin10.3 | . | √ | . | . |
| RM7276 | 10 | 14.4 | 6096007-6096157 | Bin10.4 | √ | . | . | . |
| RM1126 | 10 | 17.6 | 9294421-9294444 | Bin10.5 | √ | . | . | . |
| RM311 | 10 | 25.2 | 9747442-9747605 | Bin10.5 | . | √ | . | . |
| RM4455 | 10 | 21.8 | 11221543-11221582 | Bin10.6 | . | . | √ | √ |
| RM1859 | 10 | 26.1 | 13485257-13485443 | Bin10.7 | √ | . | √ | √ |
| RM6124 | 10 | 30.2 | 13081895-13081918 | Bin10.7 | . | . | . | √ |
| RM1375 | 10 | 42.7 | 16202087-16202148 | Bin10.9 | . | √ | . | . |
| RM3229 | 10 | 44 | 16248888-16248911 | Bin10.9 | . | . | . | √ |
| RM1873 | 10 | 48.4 | 17379879-17379914 | Bin10.9 | . | . | √ | √ |
| RM5274 | 10 | 48.4 | 17403868-17403901 | Bin10.9 | . | . | . | √ |
| RM1937 | 10 | 48.4 | 17483480-17483517 | Bin10.9 | √ | . | . | . |
| RM304 | 10 | 73 | 18211874-18211933 | Bin10.10 | . | √ | . | . |
| RM5629 | 10 | 53.6 | 18230457-18230489 | Bin10.10 | . | . | √ | . |
| RM1108 | 10 | 55.3 | 18716363-18716386 | Bin10.10 | √ | . | √ | . |
| RM1146 | 10 | 57.5 | 19169081-19169104 | Bin10.10 | √ | . | √ | √ |
| RM3773 | 10 | 58.9 | 19447567-19447602 | Bin10.10 | √ | . | . | . |
| RM6691 | 10 | 61.4 | 19785188-19785271 | Bin10.10 | . | . | √ | . |
| RM4477 | 10 | 71.4 | 20655692-20655785 | Bin10.11 | √ | . | . | √ |
| RM5352 | 10 | 71.4 | 20672962-20672987 | Bin10.11 | . | . | √ | . |
| RM1761 | 11 | 1.4 | 305169-305188 | Bin11.1 | √ | . | . | . |
| RM286 | 11 | 0 | 383875-383916 | Bin11.1 | . | √ | . | . |
| RM2459 | 11 | 10.3 | 2391067-2391096 | Bin11.2 | √ | . | √ | . |
| RM1812 | 11 | 10.3 | 2392086-2392117 | Bin11.2 | . | . | . | √ |
| RM1124 | 11 | 19.8 | 3835657-3835680 | Bin11.2 | . | √ | . | . |
| RM3133 | 11 | 32.7 | 6109517-6109544 | Bin11.4 | . | . | √ | √ |
| RM3625 | 11 | 34.8 | 6591206-6591231 | Bin11.4 | . | . | √ | . |
| RM3701 | 11 | 45.3 | 8024726-8024755 | Bin11.5 | √ | . | √ | √ |
| RM202 | 11 | 54 | 8908399-8908446 | Bin11.5 | . | √ | . | . |
| RM4862 | 11 | 54.3 | 9884968-9885023 | Bin11.5 | √ | . | . | √ |
| RM4746 | 11 | 64.2 | 16455430-16455465 | Bin11.9 | . | . | √ | . |
| RM1355 | 11 | 68.4 | 17076271-17076318 | Bin11.9 | √ | . | √ | √ |
| RM209 | 11 | 73.9 | 17808335-17808461 | Bin11.9 | . | √ | . | . |
| RM6680 | 11 | 71.4 | 18416909-18416962 | Bin11.10 | . | . | √ | √ |
| RM5349 | 11 | 79.1 | 18985185-18985205 | Bin11.10 | √ | . | √ | √ |
| RM21 | 11 | 85.7 | 19172832-19173011 | Bin11.10 | . | √ | . | . |
| RM1341 | 11 | 80.2 | 19439076-19439119 | Bin11.10 | √ | . | . | . |
| RM206 | 11 | 102.9 | 21626840-21626905 | Bin11.12 | . | √ | . | . |
| RM2191 | 11 | 101.9 | 24272058-24272101 | Bin11.13 | . | . | √ | √ |
| RM7654 | 11 | 115.1 | 26789645-26789680 | Bin11.14 | . | . | √ | . |
| RM224 | 11 | 120.1 | 26796502-26796522 | Bin11.14 | . | √ | . | . |
| RM7443 | 11 | 117.9 | 28322969-28322996 | Bin11.14 | √ | . | . | . |
| RM6973 | 12 | 49.3 | 692001-692186 | Bin12.1 | √ | . | √ | √ |
| RM3323 | 12 | 9.7 | 976022-976049 | Bin12.1 | √ | . | √ | . |
| RM3483 | 12 | 10 | 1611861-1611904 | Bin12.1 | . | √ | . | . |
| RM3747 | 12 | 12.2 | 2304482-2304515 | Bin12.2 | √ | . | √ | . |
| RM19 | 12 | 20.9 | 2432426-2432674 | Bin12.2 | . | √ | . | . |
| RM247 | 12 | 32.3 | 3185544-3185599 | Bin12.2 | . | √ | . | . |
| RM6296 | 12 | 26.7 | 3200705-3200728 | Bin12.2 | √ | . | √ | √ |
| RM6998 | 12 | 38.1 | 4745216-4745251 | Bin12.3 | √ | . | √ | . |
| RM101 | 12 | 49.5 | 8828359-8828408 | Bin12.4 | . | √ | . | . |
| RM277 | 12 | 57.2 | 18290503-18290649 | Bin12.10 | . | √ | . | . |
| RM1986 | 12 | 73 | 21282462-21282499 | Bin12.11 | √ | . | √ | √ |
| RM7018 | 12 | 75.8 | 22230170-22230197 | Bin12.12 | . | √ | . | . |
| RM3813 | 12 | 86.5 | 23313619-23313660 | Bin12.12 | √ | . | . | . |
| RM270 | 12 | 91.3 | 24969001-24969131 | Bin12.13 | . | √ | . | . |
| RM1300 | 12 | 100.9 | 26032594-26032629 | Bin12.14 | √ | . | √ | √ |
| RM2197 | 12 | 109.2 | 27420484-27420529 | Bin12.14 | √ | . | √ | . |

^1^ Genetic distance and physical position of SSR marker is from GRAMENE database (Version 39, http://www.gramene.org);

^2^ Each bin roughly represents a ~2-Mb physical distance based on the IRGSP-1.0 (http://rapdb.dna.affrc.go.jp/);

^3^ “√” indicates a polymophic marker between recurrent parent C418 and the donor parent of this column, and this marker is used for genotyping in this population; “.” indicates there is no polymorphism between the two parents of this population.

**Table S2** Detailed information on linkage disequilibrium (LD) analysis of the perfect association groups (AGs) for seedling cold tolerance detected in ILs selected from four BC_2_F_2_ populations between C418 and four different *indica* donors

| Population (code)^1^ | *AG* | **N**^2^ | **m** | ***r*** | Functional (donor) genotypic freq. | D’ | P |
| --- | --- | --- | --- | --- | --- | --- | --- |
| C418/ZH100 (A) | *AG_A1_* | 6 | 5 | 4 | 0.833 | 1.00 | 0.0005 |
|  | *AG_A2_* | 6 | 4 | 2 | 0.667 | 1.00 | 0.0005 |
| C418/Bg300 (B) | *AG_B2_* | 6 | 5 | 4 | 0.833 | 1.00 | 0.0005 |
|  | *AG_B3_* | 6 | 5 | 3 | 0.833 | 1.00 | 0.0005 |
|  | *AG_B4_* | 6 | 4 | 7 | 0.667 | 1.00 | 0.0005 |
| C418/Cis (C) | *AG_C1_* | 13 | 11 | 2 | 0.846 | 1.00 | 0.0000 |
|  | *AG_C2_* | 13 | 10 | 3 | 0.769 | 1.00 | 0.0000 |
|  | *AG_C3_* | 13 | 10 | 2 | 0.769 | 1.00 | 0.0000 |
|  | *AG_C4_* | 13 | 8 | 2 | 0.615 | 1.00 | 0.0000 |
| C418/MNTH (D) | *AG_D2_* | 5 | 4 | 6 | 0.800 | 1.00 | 0.0016 |

^1^ ZH100, Cis, and MNTH reqpresnt Zi-Hui100, Cisanggarung, and Manawthukha, respectively;

^2^ **N, m** and ***r*** represent the number of ILs, the number of individuals with co-introgression, and the number of unlinked loci involved, respectively.

**Table S3** Summarized results of 108 pairwise LD analyses between identified FGUs, either individual loci of excess introgression or perfect AGs, for seedling cold tolerance (CT) detected in 30 cold-tolerant ILs from four BC_2_F_2_ between C418 and four different donors

| Population (code) | FGU i | Branch i^1^ | FGU j | Branch j | P | D’ |
| --- | --- | --- | --- | --- | --- | --- |
| C418/ZH100 (A) | *AG_A1_* | A-I | Bin7.9 | A-I | 0.0285 | 1.00 |
|  | *AG_A2_* | A-II | Bin7.12 | A-II | 0.0285 | 1.00 |
|  | Bin7.9 | A-I | Bin7.12 | A-II | 0.2733 | -1.00 |
|  | *AG_A1_* | A-I | Bin7.12 | A-II | 0.4884 | -1.00 |
|  | *AG_A1_* | A-I | AG_A2_ | A-II | 0.2733 | -1.00 |
|  | Bin7.9 | A-I | AG_A2_ | A-II | 0.0833 | -1.00 |
| C418/Bg300 (B) | AG_B2_ | B-I | AG_B3_ | B-II | 0.4884 | -1.00 |
|  | AG_B2_ | B-I | AG_B4_ | B-I/II | 0.0285 | 1.00 |
|  | AG_B2_ | B-I | Bin8.14 | B-II | 0.2733 | -1.00 |
|  | AG_B2_ | B-I | Bin12.2 | B-I | 0.0285 | 1.00 |
|  | AG_B3_ | B-II | AG_B4_ | B-I/II | 0.0285 | 1.00 |
|  | AG_B3_ | B-II | Bin8.14 | B-II | 0.0285 | 1.00 |
|  | AG_B3_ | B-II | Bin12.2 | B-I | 0.2733 | -1.00 |
|  | AG_B4_ | B-I/II | Bin8.14 | B-II | 0.3865 | 0.25 |
|  | AG_B4_ | B-I/II | Bin12.2 | B-I | 0.3865 | 0.25 |
|  | Bin8.14 | B-II | Bin12.2 | B-I | 0.3865 | 0.25 |
| C418/Cis (C) | AG_C1_ | C-I | AG_C2_ | C-II/III | 0.2337 | -1.00 |
|  | AG_C1_ | C-I | AG_C3_ | C-III | 0.1647 | 0.35 |
|  | AG_C1_ | C-I | AG_C4_ | C-II/III | 0.6061 | 0.19 |
|  | AG_C1_ | C-I | Bin12.1 | C-I/II/III | 0.0011 | 1.00 |
|  | AG_C1_ | C-I | Bin2.18 | C-I/II/III | 0.0060 | 1.00 |
|  | AG_C1_ | C-I | Bin6.3 | C-II | 0.5302 | -1.00 |
|  | AG_C1_ | C-I | Bin4.17 | C-II | 0.1647 | 0.35 |
|  | AG_C1_ | C-I | Bin4.12 | C-II | 0.8668 | 0.07 |
|  | AG_C1_ | C-I | Bin11.10 | C-II/III | 0.0856 | -1.00 |
|  | AG_C1_ | C-I | Bin3.2 | C-III | 0.5302 | -1.00 |
|  | AG_C1_ | C-I | Bin3.18 | C-II/III | 0.0856 | -1.00 |
|  | AG_C1_ | C-I | Bin5.1 | C-III | 0.6061 | 0.19 |
|  | AG_C1_ | C-I | Bin2.4 | C-III | 0.0856 | -1.00 |
|  | AG_C2_ | C-II/III | AG_C3_ | C-III | 0.0039 | 0.57 |
|  | AG_C2_ | C-II/III | AG_C4_ | C-II/III | 0.0004 | 1.00 |
|  | AG_C2_ | C-II/III | Bin12.1 | C-I/II/III | 0.0298 | 0.52 |
|  | AG_C2_ | C-II/III | Bin2.18 | C-I/II/III | 0.1054 | 0.46 |
|  | AG_C2_ | C-II/III | Bin6.3 | C-II | 0.0072 | 1.00 |
|  | AG_C2_ | C-II/III | Bin4.17 | C-II | 0.4966 | 0.13 |
|  | AG_C2_ | C-II/III | Bin4.12 | C-II | 0.2505 | 0.38 |
|  | AG_C2_ | C-II/III | Bin11.10 | C-II/III | 0.1054 | 0.46 |
|  | AG_C2_ | C-II/III | Bin3.2 | C-III | 0.0072 | 1.00 |
|  | AG_C2_ | C-II/III | Bin3.18 | C-II/III | 0.0626 | 1.00 |
|  | AG_C2_ | C-II/III | Bin5.1 | C-III | 0.0626 | 1.00 |
|  | AG_C2_ | C-II/III | Bin2.4 | C-III | 0.1054 | 0.46 |
|  | AG_C3_ | C-III | AG_C4_ | C-II/III | 0.0004 | 1.00 |
|  | AG_C3_ | C-III | Bin12.1 | C-I/II/III | 0.0298 | 0.52 |
|  | AG_C3_ | C-III | Bin2.18 | C-I/II/III | 0.1054 | 0.46 |
|  | AG_C3_ | C-III | Bin6.3 | C-II | 0.4201 | -1.00 |
|  | AG_C3_ | C-III | Bin4.17 | C-II | 0.1261 | -1.00 |
|  | AG_C3_ | C-III | Bin4.12 | C-II | 0.4726 | -0.28 |
|  | AG_C3_ | C-III | Bin11.10 | C-II/III | 0.7685 | -0.13 |
|  | AG_C3_ | C-III | Bin3.2 | C-III | 0.0072 | 1.00 |
|  | AG_C3_ | C-III | Bin3.18 | C-II/III | 0.7685 | -0.13 |
|  | AG_C3_ | C-III | Bin5.1 | C-III | 0.0004 | 1.00 |
|  | AG_C3_ | C-III | Bin2.4 | C-III | 0.1054 | 0.46 |
|  | AG_C4_ | C-II/III | Bin12.1 | C-I/II/III | 0.0107 | 0.59 |
|  | AG_C4_ | C-II/III | Bin2.18 | C-I/II/III | 0.0743 | 0.35 |
|  | AG_C4_ | C-II/III | Bin6.3 | C-II | 0.0626 | 1.00 |
|  | AG_C4_ | C-II/III | Bin4.17 | C-II | 0.7685 | -0.13 |
|  | AG_C4_ | C-II/III | Bin4.12 | C-II | 0.6188 | -0.13 |
|  | AG_C4_ | C-II/III | Bin11.10 | C-II/III | 0.8986 | 0.03 |
|  | AG_C4_ | C-II/III | Bin3.2 | C-III | 0.0626 | 1.00 |
|  | AG_C4_ | C-II/III | Bin3.18 | C-II/III | 0.0743 | 0.35 |
|  | AG_C4_ | C-II/III | Bin5.1 | C-III | 0.0006 | 0.68 |
|  | AG_C4_ | C-II/III | Bin2.4 | C-III | 0.0743 | 0.35 |
|  | Bin12.1 | C-I/II/III | Bin2.18 | C-I/II/III | 0.0000 | 1.00 |
|  | Bin12.1 | C-I/II/III | Bin6.3 | C-II | 0.0272 | 1.00 |
|  | Bin12.1 | C-I/II/III | Bin4.17 | C-II | 0.0298 | 0.52 |
|  | Bin12.1 | C-I/II/III | Bin4.12 | C-II | 0.0492 | 0.54 |
|  | Bin12.1 | C-I/II/III | Bin11.10 | C-II/III | 0.4201 | 0.19 |
|  | Bin12.1 | C-I/II/III | Bin3.2 | C-III | 0.0272 | 1.00 |
|  | Bin12.1 | C-I/II/III | Bin3.18 | C-II/III | 0.4201 | 0.19 |
|  | Bin12.1 | C-I/II/III | Bin5.1 | C-III | 0.4201 | 0.19 |
|  | Bin12.1 | C-I/II/III | Bin2.4 | C-III | 0.3469 | -0.35 |
|  | Bin2.18 | C-I/II/III | Bin6.3 | C-II | 0.0626 | 1.00 |
|  | Bin2.18 | C-I/II/III | Bin4.17 | C-II | 0.0004 | 1.00 |
|  | Bin2.18 | C-I/II/III | Bin4.12 | C-II | 0.0062 | 0.63 |
|  | Bin2.18 | C-I/II/III | Bin11.10 | C-II/III | 0.8986 | 0.03 |
|  | Bin2.18 | C-I/II/III | Bin3.2 | C-III | 0.0626 | 1.00 |
|  | Bin2.18 | C-I/II/III | Bin3.18 | C-II/III | 0.8986 | 0.03 |
|  | Bin2.18 | C-I/II/III | Bin5.1 | C-III | 0.8986 | 0.03 |
|  | Bin2.18 | C-I/II/III | Bin2.4 | C-III | 0.1261 | -0.48 |
|  | Bin6.3 | C-II | Bin4.17 | C-II | 0.0072 | 1.00 |
|  | Bin6.3 | C-II | Bin4.12 | C-II | 0.1119 | 1.00 |
|  | Bin6.3 | C-II | Bin11.10 | C-II/III | 0.0626 | 1.00 |
|  | Bin6.3 | C-II | Bin3.2 | C-III | 0.6709 | -1.00 |
|  | Bin6.3 | C-II | Bin3.18 | C-II/III | 0.0626 | 1.00 |
|  | Bin6.3 | C-II | Bin5.1 | C-III | 0.2446 | -1.00 |
|  | Bin6.3 | C-II | Bin2.4 | C-III | 0.2446 | -1.00 |
|  | Bin4.17 | C-II | Bin4.12 | C-II | 0.0026 | 1.00 |
|  | Bin4.17 | C-II | Bin11.10 | C-II/III | 0.7685 | -0.13 |
|  | Bin4.17 | C-II | Bin3.2 | C-III | 0.4201 | -1.00 |
|  | Bin4.17 | C-II | Bin3.18 | C-II/III | 0.7685 | -0.13 |
|  | Bin4.17 | C-II | Bin5.1 | C-III | 0.0272 | -1.00 |
|  | Bin4.17 | C-II | Bin2.4 | C-III | 0.0272 | -1.00 |
|  | Bin4.12 | C-II | Bin11.10 | C-II/III | 0.2629 | 0.26 |
|  | Bin4.12 | C-II | Bin3.2 | C-III | 0.1119 | 1.00 |
|  | Bin4.12 | C-II | Bin3.18 | C-II/III | 0.2629 | 0.26 |
|  | Bin4.12 | C-II | Bin5.1 | C-III | 0.6188 | -0.13 |
|  | Bin4.12 | C-II | Bin2.4 | C-III | 0.2629 | 0.26 |
|  | Bin11.10 | C-II/III | Bin3.2 | C-III | 0.0626 | 1.00 |
|  | Bin11.10 | C-II/III | Bin3.18 | C-II/III | 0.0743 | 0.35 |
|  | Bin11.10 | C-II/III | Bin5.1 | C-III | 0.1261 | -0.48 |
|  | Bin11.10 | C-II/III | Bin2.4 | C-III | 0.8986 | 0.03 |
|  | Bin3.2 | C-III | Bin3.18 | C-II/III | 0.0626 | 1.00 |
|  | Bin3.2 | C-III | Bin5.1 | C-III | 0.0626 | 1.00 |
|  | Bin3.2 | C-III | Bin2.4 | C-III | 0.0626 | 1.00 |
|  | Bin3.18 | C-II/III | Bin5.1 | C-III | 0.0743 | 0.35 |
|  | Bin3.18 | C-II/III | Bin2.4 | C-III | 0.0743 | 0.35 |
|  | Bin5.1 | C-III | Bin2.4 | C-III | 0.0006 | 0.68 |
| C418/MNTH (D) | *AG_D2_* | D-I | Bin5.12 | D-II | 0.4292 | -1.00 |

^1^ Branch code is the same as the code showing in Figure 2.

**Table S4** Previously reported CT epistatic QTL intervals consistent with the relationships among FGUs of putative CT genetic networks detected in this study

| Epistatic QTL interval^1^ | Reference | Population | Involved AG or branch | Bins in the same AG or genetic branch |
| --- | --- | --- | --- | --- |
| RM7-RM251 vs RM225-RM204 | [12] | Bg300 | AG_B3_ | Bin 3.5 vs Bin 6.3 |
| RM225-RM204 vs RM278-OSR28 | [12] | Bg300 | AG_B3_ | Bin 6.3 vs Bin 9.10 |
| RM348-RM317 vs RM1155-RM551 | [13] | Cis | C-II | Bin 4.12 vs Bin 4.17 |
| RM1155-RM551 vs RM484-RM496 | [13] | Cis | C-I/II | Bin 4.17 vs Bin 10.10 |
| RM436-RM125 vs RM287-RM536 | [13] | Cis | C-II/III | Bin7.2 vs Bin 11.10 |
| RM528-RM340 vs RM278-RM3919B | [16] | MNTH | D-I | Bin 6.13 vs Bin 9.10 |

^1^ Physical positions of SSR markers are from the GRAMENE database (Version 39, http://www.gramene.org).

**Table S5** Signal transducers, transcription factors, and responsive proteins related to CT near or within regions of the FGUs detected in this study

| Gene name | Gene ID^1^ | Function classification | Population | FGUs involved | Branch involved | Putative upstream FGUs |
| --- | --- | --- | --- | --- | --- | --- |
| OsMEK1 | LOC_Os01g32660 | signal transductor | ZH100 | Bin 1.11 (AG_A1_) | A-I | Bin 7.1 |
| OsMAPK5 | LOC_Os03g17700 | signal transductor | Bg300 | Bin 3.5 (AG_B3)_ | B-II | AG_B1_ |
| OsCIPK15 | LOC_Os11g02240 | signal transductor | MNTH | Bin 11.10 (AG_D2_) | D-I | AG_D1_ (Bin 9.9) |
| OsCIPK14 | LOC_Os12g02200 | signal transductor | Bg300, Cis | Bin 12.1-12.2 | B-I,C-II/III | AG_B2_, **Bin 6.3** |
| OsMYB4 | LOC_Os01g50110 | Transcription factor | ZH100 | Bin 1.15 (AG_A1_) | A-I | Bin 7.1 |
| SNAC1 | LOC_Os03g60080 | Transcription factor | Cis | Bin 3.18 | C-II/III | **Bin 6.3** / Bin 3.2 |
| AP37 | LOC_Os05g41780 | Transcription factor | MNTH | Bin 5.12 | D-II | AG_D1_ (Bin 9.9) |
| OsRAN2 | LOC_Os05g49890 | Transcription factor | Bg300 | Bin 5.15 (AG_B4_) | B-I/II | AG_B2_ / AG_B3_ (**Bin 6.3**) |
| OsDREB1D | LOC_Os06g06970 | Transcription factor | ZH100, Bg300, Cis | Bin 6.3 (AG_A2_, AG_B3_) | A-II,B-II,C-II | AG_B1_, Bin 7.1-7.2 |
| OsiSAP8 | LOC_Os06g41010 | Transcription factor | Bg300, MNTH | Bin 6.12 (AG_B4_, AG_D2_) | B-I/II,D-II | AG_B2_ / AG_B3_ (**Bin 6.3**), AG_D1_ (Bin 9.9) |
| OsiSAP1 | LOC_Os09g31200 | Transcription factor | Bg300, MNTH | Bin 9.9 (AG_B3_, AG_D1_) | B-II,D-I/II | AG_B1_ |
| OsDREB1A | LOC_Os09g35030 | Transcription factor | Bg300, MNTH | Bin 9.9 (AG_B3_, AG_D1_) | B-II,D-I/II | AG_B1_ |
| OsDREB1B | LOC_Os09g35010 | Transcription factor | Bg300, MNTH | Bin 9.9 (AG_B3_, AG_D1_) | B-II,D-I/II | AG_B1_ |
| COR_1 | LOC_Os02g52820 | cold-induced protein, putative, expressed | Cis | Bin 2.18 | C-II/III | **Bin 6.3** / Bin 3.2 |
| COR_2 | LOC_Os03g55850 | cold acclimation protein WCOR413, putative, expressed | Cis | Bin 3.16 (AG_C2_) | C-II/III | **Bin 6.3** / Bin 3.2 |
| COR_3 | LOC_Os05g49170 | cold acclimation protein WCOR413, putative, expressed | Bg300 | Bin 5.15 (AG_B4_) | B-I/II | AG_B2_ / AG_B3_ (**Bin 6.3**) |
| COR_4 | LOC_Os01g18390 | Putative low temperature and salt responsive protein | Bg300 | Bin 1.5 (AG_B4_) | B-I/II | AG_B2_ / AG_B3_ (**Bin 6.3**) |
| COR_5 | LOC_Os03g25460 | Putative low temperature and salt responsive protein, expressed | Cis | Bin 3.8 (AG_C2_) | C-II/III | **Bin 6.3** / Bin 3.2 |
| COR_6 | LOC_Os05g03130 | Putative low temperature and salt responsive protein, expressed | Cis | Bin 5.1 | C-III | AG_C3_ |
| COR_7 | LOC_Os06g44220 | Putative low temperature and salt responsive protein | ZH100, Cis | Bin 6.15 (AG_A2_,AG_C1_) | A-II,C-I | Bin 7.2 |

^1^ Information of CT related genes is from the Rice Genome Annotation Project data libraries ([http://rice.plantbiology.msu.edu](http://rice.plantbiology.msu.edu/))
