# Supplementary figures and images for: Genome-wide response to selection and genetic basis of cold tolerance in rice (Oryza sativa L.)
Source: BMC Genet. 2014 May 8;15:55. doi: 10.1186/1471-2156-15-55 (PMC4024214; doi:10.1186/1471-2156-15-55)

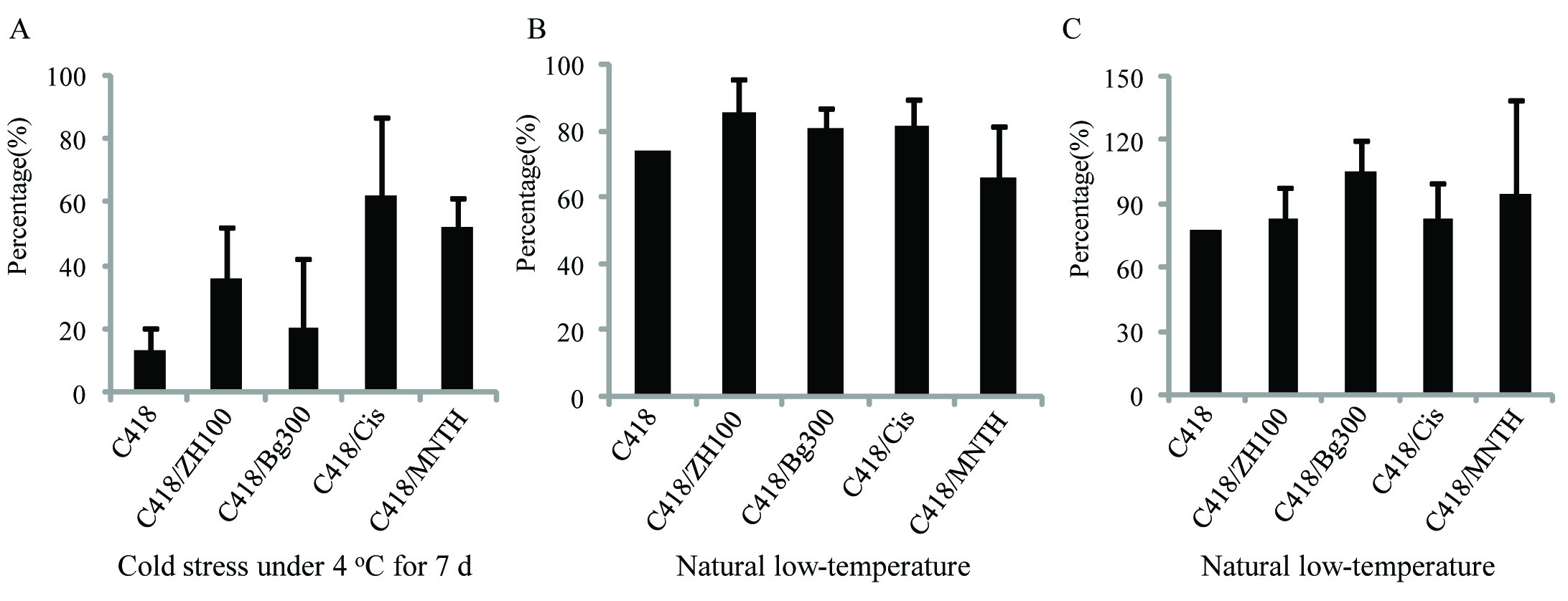

Supplement: Additional file 2: Figure S1 — Seedling cold-tolerance phenotypes of introgression lines derived from four recurrent parent C418 and four BC2F2 populations with four different indica donors. Treatments consisted of exposure to 4°C for 7 d in a growth chamber (A) and natural low-temperature conditions (B and C). (A) Seedling survival percentage. (B) Cold tolerance index of seedling height. (C) Cold tolerance index of seedling dry weight. [file 1471-2156-15-55-S2.jpeg]
